# miR-100a-5p-enriched exosomes derived from mesenchymal stem cells enhance the anti-oxidant effect in a Parkinson’s disease model via regulation of Nox4/ROS/Nrf2 signaling

**DOI:** 10.1186/s12967-023-04638-x

**Published:** 2023-10-24

**Authors:** Songzhe He, Qiongqiong Wang, Liankuai Chen, Yusheng Jason He, Xiaofang Wang, Shaogang Qu

**Affiliations:** 1 Department of Neurology, Nanfang Hospital, Southern Medical University, Guangzhou, 510515 Guangdong China; 2 Guangdong-Hong Kong-Macao Greater Bay Area Center for Brain Science and Brain-Inspired Intelligence, Guangzhou, 510515 Guangdong China; 3 Key Laboratory of Mental Health of the Ministry of Education, Southern Medical University, Guangzhou, 510515 Guangdong China; 4 ImStem Biotechnology, Inc., 400 Farmington Avenue R1808, Farmington, CT 06030 USA; 5 Zhuhai Hengqin ImStem Biotechnology Co., Ltd, Hengqin New District Huandao Donglu 1889 Building 3, Zhuhai, 519000 Guangdong China; 6 Department of Neurology, Ganzhou Hospital-Nanfang Hospital, Southern Medical University, Ganzhou, 341000 Jiangxi China

**Keywords:** Parkinson's disease, Dopaminergic neuron, Mesenchymal stem cell, Exosomes, miR-100-5p, *NOX4*

## Abstract

**Background:**

The pathogenesis of Parkinson's disease (PD) has not been fully elucidated, and there are no effective disease-modifying drugs for the treatment of PD. Mesenchymal stem cells have been used to treat several diseases, but are not readily available.

**Methods:**

Here, we used phenotypically uniform trophoblast stage-derived mesenchymal stem cells (T-MSCs) from embryonic stem cells, which are capable of stable production, and their exosomes (T-MSCs-Exo) to explore the molecular mechanisms involved in dopaminergic (DA) neuron protection in PD models using experimental assays (e.g., western blotting, immunofluorescence and immunohistochemistry staining).

**Results:**

We assessed the levels of DA neuron injury and oxidative stress in MPTP-induced PD mice and MPP^+^-induced MN9D cells after treating them with T-MSCs or T-MSCs-Exo. Furthermore, T-MSCs-Exo miRNA sequencing analysis revealed that miR-100-5p-enriched T-MSCs-Exo directly targeted the 3′ UTR of *NOX4*, which could protect against the loss of DA neurons, maintain nigro-striatal system function, ameliorate motor deficits, and reduce oxidative stress via the Nox4-ROS-Nrf2 axis in PD models.

**Conclusions:**

The study suggests that miR-100-5p-enriched T-MSCs-Exo may be a promising biological agent for the treatment of PD.

**Graphical Abstract:**

Schematic summary of the mechanism underlying the neuroprotective actions of T-MSCs-Exo in PD. T-MSCs Exo may inhibit the expression level of the target gene *NOX4* by delivering miR-100-5p, thereby reducing ROS production and alleviating oxidative stress via the Nox4-ROS-Nrf2 axis, thus improving DA neuron damage in PD.

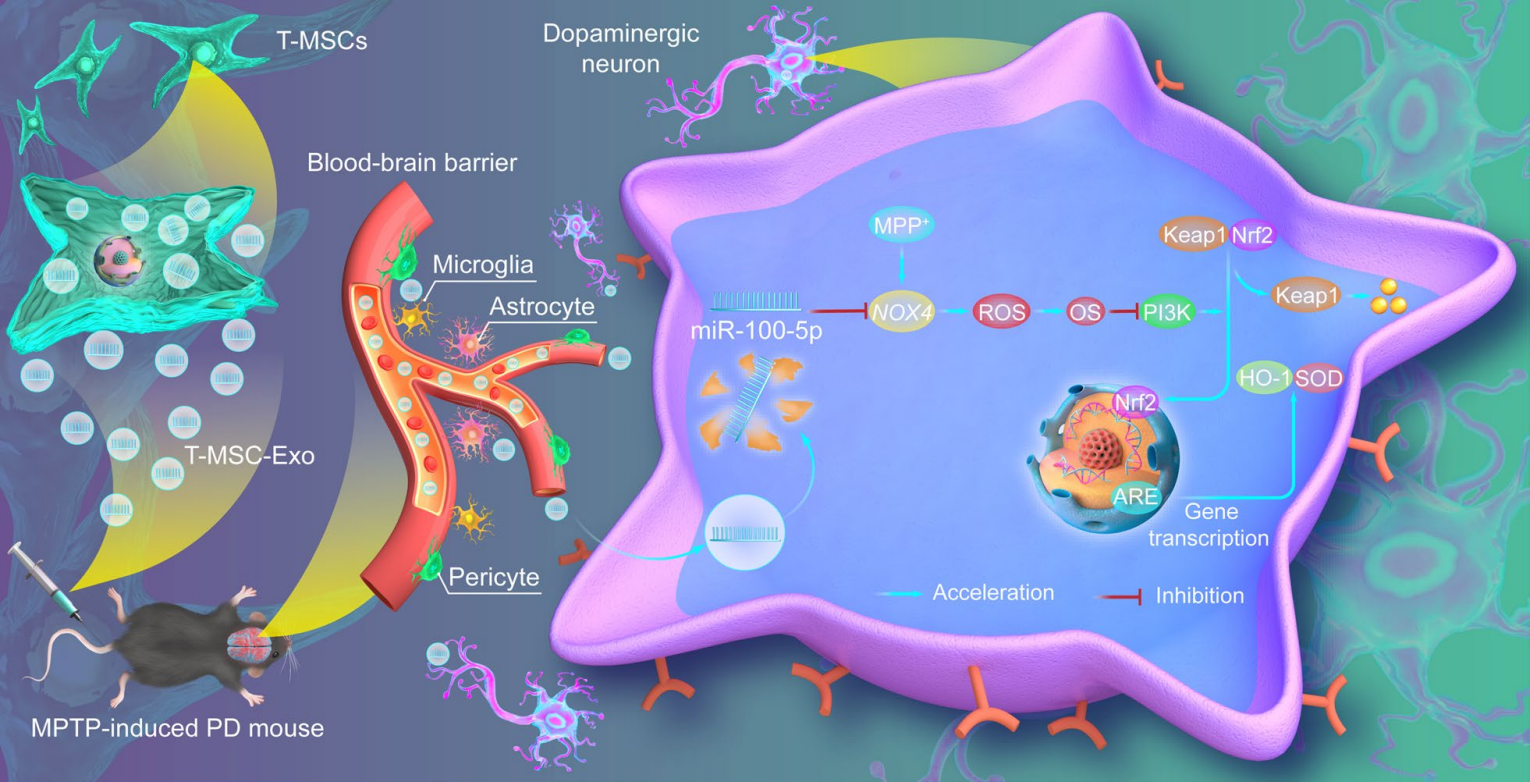

**Supplementary Information:**

The online version contains supplementary material available at 10.1186/s12967-023-04638-x.

## Introduction

Parkinson's disease (PD) is the prevalent movement disorder globally and ranks as the second most frequent age-related neurodegenerative condition. It is characterized by bradykinesia, tremor, rigidity and postural instability [1, 2]. The pathological hallmark of PD is the progressive loss of dopaminergic (DA) neurons in substantia nigra pars compacta (SNpc), accompanied by reduced DA striatal afferents, and the accumulation of misfolded α-synuclein (α-syn) in Lewy bodies. Although the pathology of PD has not been fully elucidated, compelling evidence from epidemiological, clinical, and experimental studies suggests that multiple factors (including mitochondrial dysfunction, oxidative stress, and neuroinflammation) are involved in the pathological mechanisms of PD [3–5]. With increasing life expectancy worldwide, the number of PD patients is predicted to double from 6.2 million in 2015 to 12.9 million by 2040, leading to an increasingly heavy social and economic burden [6]. To date, PD treatment including medications (e.g., levodopa) and surgery (e.g., deep brain stimulation) can only improve the clinical symptoms and does not stop disease progression to achieve a cure [7]. In view of this, it is crucial to develop new therapeutic drugs that have disease-altering properties in order to enhance the quality of life for individuals with PD.

Mesenchymal stem cells (MSCs) are nonhematopoietic, pluripotent stromal cells obtained from several tissues including bone marrow, adipose tissue, and the umbilical cord, with the capacity of self-renewal, immunomodulation, anti-inflammatory, and tissue repair effects [8, 9]. In recent years, MSCs have been utilized in the management of various neurodegenerative diseases, including PD [10]. The infusion of MSCs in a rat model of PD improved limb coordination in behavioral tests [11]. Similarly, intranasal administration of umbilical cord mesenchymal stem cells (UC-MSCs) significantly alleviated the locomotor deficits and rescued DA neurons in MPTP-induced PD mice by cross-talk between the brain and gut [12]. Furthermore, exosomes isolated from Ginkgolide A (GA) pre-treated Wharton’s jelly-derived mesenchymal stem cells (WJMSCs) significantly rescued 6-OHDA-induced SH-SY5Y cell death, reduced apoptosis-associated proteins, and further improved mitochondrial dysfunction, suggesting that GA potentiates the neuroprotective effects of exosomes derived from WJMSCs [13]. In addition, a recent preclinical study from the United States showed that the concentration of allogeneic MSCs required for administration to a single patient ranged from 1 to 10 × 10^6^ intravenous allo-hMSCs/kg, and that this concentration was safe, well-tolerated, and non-immunogenic in patients with mild-to-moderate PD, suggesting that allo-hMSCs from young healthy donors are a potentially effective treatment of PD [14]. Although MSCs have been extensively applied in preclinical studies of several human diseases in recent years, their clinical application was restricted due to their limited sources and small numbers. In order to address certain drawbacks of MSCs derived from adult tissue, a special type of MSCs (trophoblast stage-derived MSCs, T-MSCs) obtained rapidly and efficiently from commercial embryonic stem cells at the trophoblast stage within 11–16 days has demonstrated effectiveness in certain models of autoimmune diseases [15, 16].

Recent studies have shown that the therapeutic effect of MSCs mainly relies on the secretion of exosomes (MSC-derived exosomes, MSC-Exo) [17, 18]. Meanwhile, compared to MSCs, MSC-Exo (with a diameter of 40–160 nm) are important bioactive extracellular vesicles with potential value for biological therapy due to their high safety, ability to cross the blood–brain barrier (BBB), and abundant sources [19]. Furthermore, there is growing evidence that MSC-Exo contain abundant contents (including nucleic acids, proteins, mRNAs, and miRNAs), which play a critical role in intercellular communication [20]. Except for controlling regular physiological functions like tissue healing [21] and immune surveillance [22], MSC-Exo also play a role in the pathological mechanisms of nervous system injury, such as PD [23, 24]. For example, downregulation of exosomal miR-137 in the serum of PD mice attenuates oxidative stress damage in PD by upregulating OXR1 [25]. Moreover, the PR-EXO/PP@Cur, a nanocarrier that prioritizes itself, effectively addresses the intricate pathology of PD by reducing neuroinflammation and enhancing the restoration of neuronal function [2]. Based on these, we explored whether T-MSCs were involved in PD-related neuroprotective effects via exosomal miRNAs.

Although the potential contribution of MSCs and their exosomes in the treatment of PD has been emphasized in previous research, it is important to acknowledge their limitations in clinical translation, such as their optimal dosage, frequency, and long-term safety and effectiveness. Additionally, assessing the long-term safety and sustained effectiveness of this innovative therapy is crucial for its successful implementation. Therefore, in the light of the current lack of disease-modifying drugs for PD, our research assessed the levels of DA neuron injury and oxidative stress in MPTP-induced PD mice and MPP^+^-induced MN9D cells after treating them with T-MSCs or T-MSCs-Exo. Using T-MSCs-Exo miRNA sequencing analysis, we screened miR-100-5p that is closely associated with PD. Further research confirmed that *NOX4* is a direct target of miR-100-5p. The data showed that overexpressed miR-100-5p had therapeutic effects in PD by suppressing oxidative stress via targeting *NOX4* both in vitro and in vivo. The present results revealed a role of T-MSCs-Exo in regulating DA neuron protection and suggested that miR-100-5p-enriched T-MSCs-Exo may be a promising biological agent for the repair of damage in PD models.

## Materials and methods

### T-MSCs culture and identification

T-MSCs were provided by ImStem Biotechnology Inc. All procedures were carried out in accordance with the National Institutes of Health Guidelines on Human Stem Cell Research. Commercial ESI − 053 embryonic stem cells were cultured in this study to derive T-MSCs. Cell surface phenotype and trilineage differentiation were performed as previously reported [15].

We induced commercial ESI-053 embryonic stem cells differentiation into trophoblast (TB)-like cells and found the TB-like cell morphology observed after 5 days. Simultaneously, the TB-like cells were enzymatically separated and transferred to gelatin-coated plates in α-MEM (Gibco, California, USA) supplemented with 10% PLTGold® Human Platelet Lysate (BI, Kibbutz Beit-Haemek, Israel), 1% MEM NEAA (Gibco, California, USA), and 1% GlutaMAX (Gibco, California, USA). The cell culture medium was changed every 48 h, and the colonies of mesenchymal-like cells (T-MSCs, passage 0) that emerged between days 11 and 16 were passaged every 7 days. T-MSCs at P3, P4, and P5 were used for the experiments, and phenotype markers (CD73, CD90, CD105, and CD31) were examined by flow cytometry before the experiments (Additional file 1: Fig. S1).

### MN9D cell culture

MN9D cells were purchased from the American Type Culture Collection (ATCC, Manassas, VA, USA) and were cultured in DMEM (Gibco, California, USA) containing 8% fetal bovine serum (Gibco, California, USA), and 1% penicillin/streptomycin/smphotericin B (Beyotime Biotechnology, Shanghai, China) in an incubator at 37 °C with 5% CO_2_. Standard cell culture techniques were employed for cell passaging once the MN9D cells achieved 80% confluency. Specifically, for routine cell culture passaging, 0.25% Trypsin–EDTA (Beyotime Biotechnology, Shanghai, China) was used to detach cells from the cell culture flask or plates until the logarithmic growth phase.

### Cell viability assay

A suspension of MN9D cells (approximately 1.5 × 10^3^ cells) was introduced into 96-well plates. Cells were co-incubated with different concentrations (100, 200, 500, 750, and 1000 μmol) of MPP^+^ (Sigma-Aldrich, St. Louis, MO, USA), followed by incubation in a 37 °C cell incubator with 5% CO_2_ for 24 h. At prespecified time points on day 2, 10 μL CCK-8 (cell counting kit-8) solution (Beyotime Biotechnology, Shanghai, China) was added to each well, after which the plates were incubated for 2 h. Optical density values at 450 nm were determined using a multifunctional microplate reader (Infinite 200 Pro, Switzerland). Each group was tested in three replicate wells and three independent experiments were performed, respectively. The relative cell viability of the experimental groups was calculated compared to the control group.

### Animals

Male C57BL/6 mice aged 6 − 8 weeks, and weighing between 22 and 25 g, were obtained from Zhejiang Vital River Laboratory Animal Technology Co., Ltd. (Jiaxing, Zhejiang, China). Mice were acclimated to the environment and food of the animal facilities for at least 1 week before experiments. Six mice per cage were maintained under a 12/12-h dark–light cycle at a relative humidity of 60 ± 5% and a constant temperature of 22 ± 1 °C. All experiments were conducted in accordance with the criteria outlined in the Guide for the Care and Use of Laboratory Animals published by the National Institutes of Health (NIH publication No. 8023, revised 1985), and were approved by the Institutional Animal Care and Use Committee of Southern Medical University.

### Experimental in vivo paradigm and drug treatment

The first set of mice were randomly divided into four groups: a control group, T-MSCs group, MPTP group, and MPTP + T-MSCs group (n = 10 per group). MPTP (25 mg/kg; Sigma-Aldrich, USA) was injected intraperitoneally (twice a week, 5 weeks) in the MPTP and MPTP + T-MSCs groups, whereas the other groups were injected intraperitoneally with an equal volume of 1 × PBS. Next, the T-MSCs and MPTP + T-MSCs groups received intravenous T-MSCs (5 × 10^5^ cells/100 μL PBS) administration on days 3, 17, and 31 after modeling, while the other groups received 1 × PBS. Following the conclusion of the treatment and analysis of behavior, the mice were euthanized and perfused intracardially with ice-cold 1 × PBS. The tissues were rapidly dissected to test the relevant indicators.

The second set of mice were randomly divided into eight groups: a control group (n = 10), T-MSCs-Exo (2.44 × 10^10^ particles/mL) group (n = 7), T-MSCs-Exo (7.32 × 10^10^ particles/mL) group (n = 7), T-MSCs-Exo (1.22 × 10^11^ particles/mL) group (n = 10), MPTP group, MPTP + T-MSCs-Exo (2.44 × 10^10^ particles/mL) group (n = 7), MPTP + T-MSCs-Exo (7.32 × 10^10^ particles/mL) group (n = 10), and MPTP + T-MSCs-Exo (1.22 × 10^11^ particles/mL) group (n = 7). MPTP (25 mg/kg; Sigma-Aldrich, USA) was injected intraperitoneally (twice a week, 5 weeks) in the MPTP and MPTP + T-MSCs-Exo groups, while the other groups were injected intraperitoneally with an equal volume of 1 × PBS. Next, for the T-MSCs-Exo and MPTP + T-MSCs-Exo groups, we isolated T-MSCs-Exo (2.44 × 10^10^ particles/mL, 7.32 × 10^10^ particles/mL, and 1.22 × 10^11^ particles/mL) in PBS and administered them respectively by intravenous injection on days 3, 17, and 31 after the start of modeling, while the other groups received 1 × PBS. After completion of the treatment and behavior analysis, the mice were euthanized and transcardially perfused with ice-cold 1 × PBS. The tissues were rapidly dissected to test the relevant indicators.

### Open field test

The mice were placed in the center of an open field chamber (40 × 40 × 40 cm) and a video monitor connected to a recording and analysis system (Shanghai, China) was attached to the chamber to record the movements and behavior of the mice for 10 min. Each chamber was wiped with 75% ethanol after each experiment. The mice’s trajectory, total distance, speed, and central area distance in the box were recorded and analyzed.

### Rotarod test

The mice underwent training on a rotarod (RotaRod Advanced, TSE, Germany) twice daily for 3 consecutive days, with each session lasting 5 min at a speed of 5 rpm. During the rotarod experiment, the speed was accelerated from 4 to 40 rpm within 5 min, while monitoring the duration of mouse movement on the rotarod.

### Hang test

In accordance with our prior research, the hang test was employed to evaluate the synchronization of limb motions [26, 27]. Each mouse was held from a wire held across the front paws with their tails fixed. The time taken for each mouse to stomp on the wire with both hind limbs or to fall was recorded. The mice were trained three times a day, at least 10 min apart, and then scored on the fourth day, and scored using the following criteria: 0 − 4 s = 0, 5 − 9 s = 1, 10 − 14 s = 2, 15 − 19 s = 3, 20 − 24 s = 4, 25 − 29 s = 5, and > 30 s = 6.

### Pole test

Mice in each group were placed on the top of a vertical wooden pole (50-cm long with a 1-cm diameter). The recorded duration of the mice’s descent from the top of the pole to the base was noted.

### Isolation and analysis of T-MSCs-Exo

According to previous reports [28], we used the polyethylene glycol (PEG) method to enrich T-MSCs-Exo containing the medium from the cell culture. After collecting the cell supernatant, it was subjected to centrifugation at 500 g for 5 min, followed by another centrifugation at 4 °C for 30 min at 2000*g* to remove dead cells, cellular debris and apoptotic bodies. After centrifugation, a solution containing 1 M NaCl and 24% (w/v) PEG6000 was mixed with the supernatant at a ratio of 1:1 to reach a final concentration of PEG6000 of 12% in the mixed solution. The abovementioned solution was kept at 4 °C for overnight precipitation, and the samples were harvested by centrifugation at 3000 g for 60 min on the following day. Subsequently, the supernatant was discarded and the tubes were inverted to settle for 5 min, with occasional tapping to eliminate any extra PEG. The pellet obtained was resuspended in 50–500 μL of 1 × PBS (pH = 7.4). Afterwards, the samples were preserved at − 80 °C. The sizes of purified T-MSCs-Exo were determined using A NanoSight NS300 (Malvern Instruments, UK) Nanoparticle Tracking Analysis (NTA) system. The bicinchoninic acid (BCA, Beyotime Biotechnology, Shanghai, China) assay was performed to measure the protein concentration in T-MSCs-Exo. In particular, we used protein standards according to the concentration gradient (0, 0.025, 0.05, 0.1, 0.2, 0.3, 0.4, and 0.5 mg/mL) in the standard wells of a 96-well plate. Then 1 × PBS was added to reach a volume of 20 µL. Meanwhile, we added 2 µL of T-MSCs-Exo into the sample wells of the 96-well plate, and make up to 20 µl with 1 × PBS. Next, we added 200 µL of the prepared BCA working solution (50:1 ratio of BCA reagent A and reagent B, mixed thoroughly) into each well and incubated them at 37ºC for 25 min. The optical density values were determined at 450 nm using a multifunctional microplate reader (Infinite 200 Pro, Switzerland). The protein concentration in T-MSCs-Exo was calculated from the standard curve. Western blotting was used to measure TSG101, CD9, HSP70, and calnexin protein levels. A transmission electron microscope (TEM) was used with a Tecnai G2 (Tecnai, USA) to analyze the T-MSCs-Exo ultrastructure.

### T-MSCs-Exo tracing

For the in vitro uptake studies, purified T-MSCs-Exo were labeled with an Exosome Staining Quick kit (H-Wayen, Shanghai, China) following the instructions provided by the manufacturer. Briefly, an equal volume of 2 × staining working solution was prepared using a 1:25 dilution of fluorescent dye buffer, and then T-MSCs-Exo were mixed at a ratio of 1:1 with the staining working solution while being protected from light for 5 min at room temperature. Next, free fluorescence was removed using a fluorescence adsorption column buffer replacement method. Finally, T-MSCs-Exo were resuspended in serum-free medium and co-cultured with MN9D cells for 1, 3, 6, 12, and 24 h. Then, the T-MSCs-Exo were fixed, subjected to DAPI staining and observed using an LSM 980 (ZEISS, Germany).

For the in vivo uptake studies, T-MSCs-Exo were labeled using the PKH26 Red Fluorescent Cell Linker Kit (MKbio, Shanghai, China) according to the manufacturer’s instructions. Then, 5 μg of PKH26-labeled T-MSCs-Exo were adoptively transferred into the normal mice via tail vein injection. Once the injection was completed, the brain enrichment of PKH26-labelled T-MSCs-Exo were examined at each time interval at 0, 2, 6, 8, 10, 12, and 48 h using the Automated In-Vivo Imaging (Caliper, USA). Afterwards, the major organs of mice (including brain, heart, liver, spleen, lungs and kidneys) were isolated by anesthetizing the mice and transcardially perfusing them pre-chilled 1 × PBS. The organ distribution of T-MSCs-Exo was detected using an optical imaging system (Caliper, USA). After completion of live imaging and the fluorescence imaging analysis of major organ distribution, brain tissues from mice at each time point were fixed, dehydrated, embedded and frozen. Then the uptake of exosomes in different cells (e.g. DA neurons, astrocytes, and microglia) was detected by immunofluorescence staining.

### Co-culture of MPP^+^-induced MN9D cells and T-MSCs

To confirm whether T-MSCs are involved in the protective effects of PD, several groups were formed including: a control group, T-MSCs group, MPP^+^ group, and MPP^+^ + T-MSCs group. The cell viability was assessed after treating MN9D cells with 500 μΜ MPP^+^ for 24 h. Then, MN9D cells were co-cultured with T-MSCs in using transwell 6-well plates (0.4 µm pore, PET membrane; LABSELECT, Hefei, China) to prevent direct cell contact for 24 h. MN9D cells were planted in the lower chambers and T-MSCs were added to the upper wells. Once the treatment was completed, the corresponding indicators were tested. All experiments were repeated three times independently.

### T-MSCs-Exo treatment of MPP^+^-induced MN9D cells

To clarify whether T-MSCs are involved in neuroprotective effects through secreted T-MSCs-Exo secretion, the following groups were established: a control group, T-MSCs-Exo group, MPP^+^ group, and MPP^+^ + T-MSCs-Exo group. MN9D cells were incubated with 500 μΜ MPP^+^ for 24 h, and treated with 50 μg/mL T-MSCs-Exo for 24 h, after which the cell viability was detected. Eventually, the cells were gathered to extract the proteins and other corresponding indicators.

GW4869 (Sigma-Aldrich,St. Louis, MO, USA) was used to reduce the release of T-MSCs-Exo from T-MSCs at a final concentration of 40 µM. Before the T-MSCs were co-cultured with MN9D cells, they were pretreated with GW4869 for 24 h. Once the treatment was completed, the corresponding indicators were tested. All experiments were repeated three times independently.

### Western blotting

Protein lysates from cells, tissues, or exosome lysates were prepared in RIPA buffer (Beyotime Biotechnology, Shanghai, China) and quantified using a BCA kit (Beyotime Biotechnology, Shanghai, China). After undergoing SDS-PAGE, the samples were transferred to PVDF membranes. Then, the PVDF membranes with the indicated protein samples were blocked with 5% non-fat milk or BSA and incubated with the primary antibody. Following incubation with the secondary antibody, chemiluminescence signals were detected using Tanon 5200 (Tanon, Shanghai, China). Protein expression was normalized to β-actin levels.

### Immunofluorescence and immunohistochemistry staining

For the immunofluorescent assay, the cell climbing slices or brain slices were incubated overnight with the respective primary antibodies at 4 °C. After that, they were thoroughly washed with 1 × PBS and subsequently treated with fluorescent-labeled secondary antibodies for 45 min. Afterwards, DAPI was added to stain the cell nuclei. Images were acquired with a confocal laser-scanning microscope LSM 980 (ZEISS, Germany). For the immunohistochemistry assay, brain slices were incubated with the corresponding primary antibody overnight at 4 °C. Following this, they were washed with 1 × PBS, exposed to a biotin-labeled secondary antibody, and finally stained using diaminobenzidine (DAB). Images were collected under a microscope (Leica SAPO, Hamburg, Germany). The Image-Pro Plus 8.0 photogram analysis system (IPP 8.0, Media Cybernetics, Bethesda, MD, USA) was utilized for conducting quantitative analysis. Each experiment was repeated three times independently.

### Reactive oxyen species (ROS) detection

Next, 2,7-dichlorofluorescein diacetate (DCFH-DA) (Beyotime Biotechnology, Shanghai, China) and Hoechst 33342 Staining (Beyotime Biotechnology, Shanghai, China) working solutions were produced to evaluate the ROS level in MN9D cells. After incubating for 10 min at 37 °C, the cells were washed three times with basal medium to effectively eliminate any remaining DCFH-DA and Hoechst 33342 staining that had not penetrated the cells. Subsequently, images were scanned under a fluorescent microscope (Olympus, Japan). Each experiment was independently repeated a minimum of three times.

### GSH and GSSG detection

The levels of GSH and GSSG were measured using the GSH/GSSG kit (Beyotime Biotechnology, Shanghai, China) according to the manufacturer’s instructions. Mouse tissues were flash-frozen in liquid nitrogen, powdered, added to the Protein Removal Reagent M solution, and ground into a homogenate. To determine the overall glutathione level, the liquid remaining after spinning at 10,000 g for 10 min was utilized. Next, in order to degrade GSH, the solution containing the reagent that scavenges GSH was introduced to the samples. Then, appropriate amounts of complete glutathione working solution were included and combined with NADPH, and the measurement of absorbance at 412 nm was conducted using a microplate reader (Infinite 200 Pro, Switzerland). The GSH concentration was obtained by subtracting the GSSG levels from the total glutathione levels. Based on these values, the GSH/GSSG ratio was calculated. Data were obtained from at least three independent experiments.

### Nrf2 inhibitor treatment

To investigate the functional roles of signaling pathways in MPP^+^-induced damage in MN9D cells, the cells were pretreated with or without 50 nM ML385 (Nrf2 inhibitor) for 2 h and then stimulated with 500 µM MPP^+^ for 24 h. Next, drug-treated (T-MSCs or T-MSCs-Exo) cells were further incubated for 24 h. The cells were subjected to detect oxidative stress-related proteins by western blotting. All experiments were repeated three times independently to ensure reproducibility.

### Sequencing analysis of T-MSCs-Exo miRNAs

The sequencing analysis was performed at OBiO Technology Inc. (Shanghai, China). The miRNA collection was created following the standard procedures outlined by Illumina, which encompassed library preparation and sequencing. Small RNA sequencing libraries were constructed using the TruSeq Small RNA Sample Prep Kits (Illumina, San Diego, USA), according to the manufacturer’s protocol. Following the preparation of the library, the libraries were sequenced using Illumina Hiseq 2000/2500 (50-bp single-end reads). Quantile normalization and subsequent data processing were performed using ACGT101-miR software (LC Sciences, Houston, Texas, USA). The miRNA target genes were predicted using miRanda and TargetScan online software analysis.

### miR-100-5p inhibitor/mimic transfection

For transient transfection, MN9D cells were transfected with miR-100-5p inhibitor, inhibitor NC, mimic, and mimic NC of *NOX4* (Genepharma, Shanghai, China). The manufacturer’s protocol was followed to perform transient transfection with lipofectamine 2000 (Invitrogen, California, USA). Their sequences are listed in Additional file 1: Table S1.

### Stereotactic injection of miR-100-5p in the substantia nigra (SN)

The mice were randomly divided into six groups: a control group (n = 10), AAV-miR-NC group (n = 8), AAV-miR-100-5p group (n = 10), MPTP group (n = 8), MPTP + AAV-miR-NC group (n = 7), and MPTP + AAV-miR-100-5p group (n = 10). Adeno-associated viruses (AAVs) overexpressing miR-100-5p were purchased from Shanghai SunBio Medical Biotechnology Co., Ltd (Shanghai, China). AAV expressing miR-100-5p (AAV-CAG-GFP-miR-100-5p-WPRE, 6.66 × 10^13^ vg/mL, CCUGUUGCCACAAACCCGUAGAUCCGAACUUGUGCUGAUUCUGCACACAAGCUUGUGUCUAUAGGUAUGUGUCUGUUAGG) or an empty vector (AAV-control) were stereotaxically injected into the left side of the SNpc at the target site (bregma AP, − 3.0 mm; ML, − 1.2 mm; DV, − 4.5 mm). Briefly, 2.0 μL of virus or AAV-control was injected stereotaxically into the SNpc. After 2 weeks, MPTP (30 mg/kg; Sigma-Aldrich, USA) or an equal volume of saline was intraperitoneally injected for 5 days. Three days after the last MPTP injection, behavioral experiments were conducted and the animals were euthanized for tissue collection.

### RNA isolation and quantitative real-time PCR (qRT-PCR)

Total RNA was extracted from cells or tissues using TRIzol reagent (Takara, Otsu, Japan) following the manufacturer’s protocol. For mRNA detection, reverse transcription was performed using PrimeScript™ RT reagent Kit with gDNA Eraser (Takara, Otsu, Japan). qRT-PCR was performed using TB Green Premix Ex Taq (Takara, Otsu, Japan) and measured on LightCycler^®^480 (Merck Group, Darmstadt, Germany). β-actin was used as the reference control, and its expression was evaluated using the standard 2^−ΔΔCt^ method. For microRNA analysis, poly (A) was added to the microRNA, and reverse transcription was performed using 4 × microRNA Reverse Transcription Mix (EZBioscience, Roseville, USA). qRT-PCR was performed using TB Green Premix Ex Taq (Takara, Otsu, Japan) and measured on a LightCycler®480 (Merck Group, Darmstadt, Germany). MicroRNA expression was normalized to U6 expression and calculated via the standard 2^−ΔΔCt^ method. The repetition of every experiment was conducted at least three independent times. The primer sequences are listed in Additional file 1: Table S2.

### Dual luciferase reporter assay

To investigate whether miR-100-5p directly targeted *NOX4*, we constructed luciferase reporter vectors using Hippobio (Huzhou, China). Next, a luciferase construct containing wild-type or mutant-binding sites for *NOX4* was transfected into MN9D cells, together with a miR-100-5p mimic or an empty vector. After transfection, luciferase activities in the cells were assessed using a Dual-Luciferase^®^ Reporter Assay kit (Promega, Sunnyvale, CA, USA) according to the manufacturer’s instructions. Each experiment was conducted at least three times with consistent results.

### Statistical analysis

All statistical analyses were conducted using GraphPad Prism 8.0 Software (GraphPad Software Inc., San Diego, CA, USA). Data are shown as means ± SD collected from at least three independent experiments. One-way ANOVA was used to assess statistical significance. *p* < 0.05 was considered statistically significant. The antibodies used in this study are listed in Additional file 1: Table S3.

## Results

### T-MSCs contribute to MPP^+^-induced MN9D cell proliferation, and improve motor deficits in PD mice

To evaluate the hypothesis that trophoblasts may have the potential to differentiate into T-MSCs, we induced the differentiation of commercial ESI-053 embryonic stem cells into TB-like cells. Additional file 1: Fig. S1a presented the TB-like cell morphology observed after 5 days. At the same time, the TB-like cells were enzymatically detached and replated on gelatin-coated plates in α-MEM (Gibco, California, USA) supplemented with 10% PLTGold® Human Platelet Lysate (BI, Kibbutz Beit-Haemek, Israel), 1% MEM NEAA (Gibco, California, USA), and 1% GlutaMAX (Gibco, California, USA). The cell culture medium was changed every 48 h, and colonies of mesenchymal-like cells (T-MSCs, passage 0) emerged between days 11 and 16, which were then passaged every 7 days (Additional file 1: Fig. S1a).

Next, for the identification of the mesenchymal-like cells in our culture, they were harvested at passage 3, and the cell phenotypes were detected using flow cytometry. The results showed that the cells were negative for CD34 expression and common MSC markers, including CD73, CD90, and CD105 (Additional file 1: Fig. S1b). Therefore, the mesenchymal-like cells used in our study met the criteria for the identification of multipotent MSCs by the guidelines proposed by the Mesenchymal and Tissue Stem Cell Committee of the International Society for Cellular Therapy (ISCT) [29]. We refer to the MSCs derived via the TB-like intermediate as T-MSCs, to distinguish them from MSCs derived from the master cell bank (MCB) through alternative differentiation methods.

To determine the specific concentration of MPP^+^ in MN9D cells, we used a series of MPP^+^ concentrations to induce MN9D cells for 24 h. MPP^+^ induced a dose-dependent decrease in cell viability, as assessed by the CCK-8 assay in MN9D cells. Meanwhile, the viability of MN9D cells decreased by more than 25% at a concentration of 500 μM, therefore this concentration was selected for subsequent studies (Additional file 1: Fig. S2a).

To further validate the effect of T-MSCs on MN9D cells, we adopted a pre-screened MPP^+^ concentration (500 μM) to construct a PD cell model. Western blotting showed that the expression of tyrosine hydroxylase (TH; rate-limiting enzyme in synthesis of dopamine) was significantly reduced in MPP^+^-induced MN9D cells. Additionally, T-MSCs prevented an MPP^+^-mediated decrease in TH expression (Additional file 1: Fig. S2b, c). These results indicate that T-MSCs can increase TH expression in MPP^+^-induced MN9D cells.

**Fig. 1:**
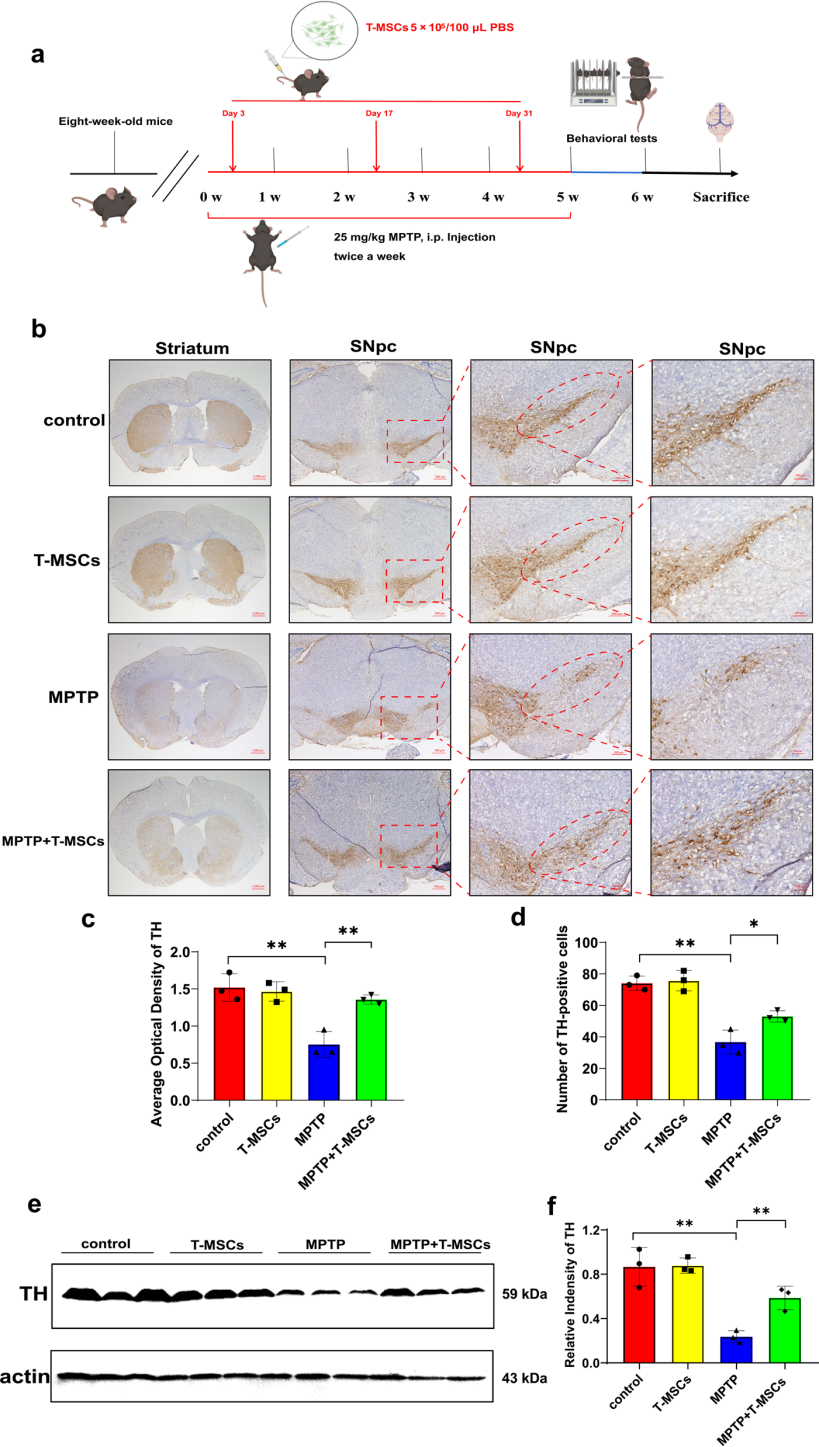
Neuroprotective effects of T-MSCs in MPTP-induced PD mice. **a** Experimental protocol schematic in vivo. **b**–**d** Representative images and quantification of IHC of TH-positive neurons in the striatum and SNpc of the control, T-MSCs, MPTP, and MPTP + T-MSCs groups (n = 3 per group). Scale bars, 1000 µm for images in striatum; 500, 200, and 100 µm for the series of images in SNpc. **e**, **f** Western blotting analysis showed the TH expression levels in the SN of the control, T-MSCs, MPTP, and MPTP + T-MSCs groups (n = 3 per group). The results are shown as mean ± SD. One-way ANOVA was used to analyze the data. *p < 0.05, **p < 0.01

Next, to evaluate whether T-MSCs may provide therapeutic efficacy for PD in vivo, we constructed a chronic PD model by intraperitoneal injection of MPTP (25 mg/kg) in C57BL/6 mice. T-MSCs (5 × 10^5^ cells/100 μL PBS) were administered in the tail vein on days 3, 17 and 31 after the start of modeling (Fig. 1a). Following the analysis of behavioral tests, compared to the control group, the MPTP group moved shorter distances, spent less time on the rotarod test, scored lower on the hang test and spent longer time on the pole test. However, T-MSCs improved the abovementioned changes in PD mice (Additional file 1: Fig. S3a–g). The abovementioned data suggest that T-MSCs can improve motor deficits in PD mice.

Then, we examined the expression of TH in the midbrain of the abovementioned four groups of mice. Immunohistochemical staining (IHC) showed that the MPTP + T-MSCs group had greater TH density and quantity of TH-positive neurons in the striatum and SNpc than the MPTP group (Fig. 1b–d). Western blotting assay showed that MPTP resulted in a significant reduction in TH expression in the midbrain of mice, while T-MSCs treatment of PD mice resulted in a significant rebound in TH expression (Fig. 1e, f). In addition, mutation, aggregation, and excessive accumulation of α-syn, a protein consisting of 140 amino acids located at the presynaptic terminus of neurons, is closely associated with a range of neurodegenerative diseases including PD. The protein results showed that MPTP induced α-syn aggregation in the midbrain of mice, while T-MSCs treatment of PD mice significantly prevented α-syn aggregation (Additional file 1: Fig. S3h, i). The abovementioned results confirm that T-MSCs alleviate MPTP-induced DA neuron damage in PD mice, prevent α-syn aggregation, and reduce the toxicity of MPTP on neurons.

### Isolation and characterization of T-MSCs-Exo and their protective effect against PD

To investigate how T-MSCs protect against PD, we used polyethylene glycol (PEG) precipitation to extract the supernatant of T-MSCs and purify it for analysis by TEM, nanosight tracking analysis (NTA), and western blotting. The results of TEM and NTA revealed that the particles isolated from the supernatant of T-MSCs contained abundant exosomes with a diameter of 123.6 ± 2.8 nm. In addition, western blotting analysis confirmed the presence of exosome specific markers (HSP70, TSG101, and CD9). The absence of calnexin further verified that the isolated particles were mainly T-MSCs-Exo, indicating that T-MSCs can secrete exosomes (Additional file 1: Fig. S4).

**Fig. 2:**
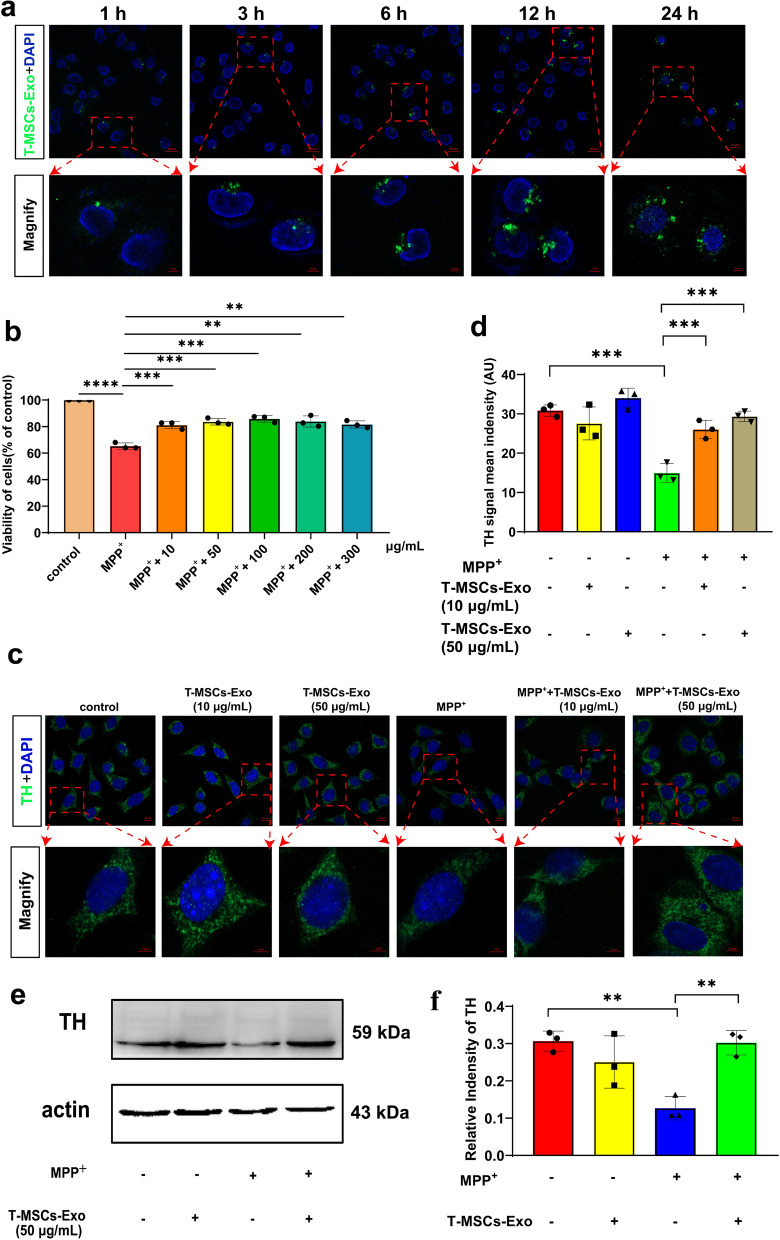
T-MSCs-Exo could be taken up by MN9D cells via endocytosis and enhanced cell viability in MPP^+^-induced MN9D cells. **a** T-MSCs-Exo were labelled using the Exosome Tracer Kit and co-cultured with MN9D cells. During this period, five time points (1, 3, 6, 12, and 24 h) were set. Cells from each time point were stained with DAPI and the uptake of T-MSCs-Exo by MN9D cells was observed by confocal microscopy. Scale bars, upper, 20 µm; lower, 5 µm. **b** CCK-8 was used to evaluate the MPP^+^-induced MN9D cell viability after treatment with 10, 50, 100, 200, and 300 μg/mL T-MSCs-Exo for 24 h. **c**, **d** Immunofluorescence staining and quantification of TH expression in MPP^+^-induced MN9D cells after treatment with different concentrations of T-MSCs-Exo for 24 h. **e**, **f** Western blotting analysis showed the TH expression on MPP^+^-induced MN9D cells after treatment with T-MSCs-Exo for 24 h. Each experiment was independently repeated three times, and the results are shown as mean ± SD. One-way ANOVA was used to analyze the data. **p < 0.01, ***p < 0.001, ****p < 0.0001

According to previous studies, the therapeutic effects of MSCs may be achieved through exosome secretion [30]. To verify whether T-MSCs-Exo could be taken up by MN9D cells, purified T-MSCs-Exo were labeled using an Exosome Staining Quick kit (H-Wayen, Shanghai, China) and co-cultured with MN9D cells. Uptake of labeled T-MSCs-Exo by MN9D cells was demonstrated using the laser scanning confocal microscopy (Fig. 2a). Furthermore, we adopted the appropriate concentrations of T-MSCs-Exo screened by the CCK8 assay method to act on MPP^+^-induced MN9D cells, and then detected TH fluorescence intensity and protein expression levels by immunofluorescence staining and western blotting (Fig. 2b–f). The results demonstrated that 50 μg/mL of T-MSCs-Exo significantly increased the TH fluorescence intensity and protein expression in MPP^+^-induced MN9D cells, so this concentration was selected for the subsequent experiments.

**Fig. 3:**
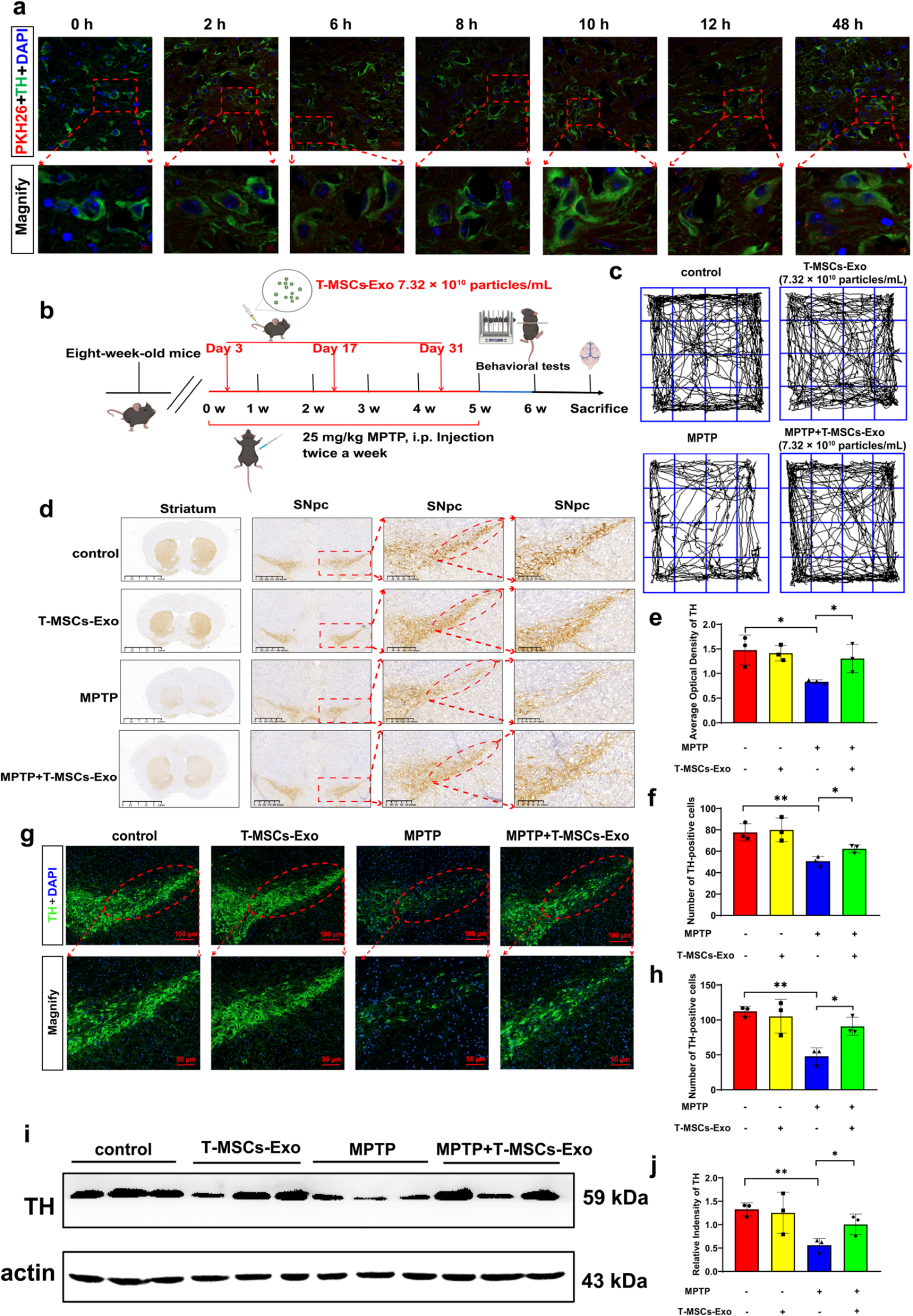
T-MSCs-Exo can cross the BBB to reach the SN and alleviate the degeneration of DA neurons in PD mice. **a** We injected PKH26-labeled T-MSCs-Exo into mice via the tail vein, and subjected them to cardiac perfusion for brain extraction at 0, 2, 6, 8, 10, 12 and 48 h. Next, we prepared frozen sections and detected the uptake of PKH26-labeled T-MSCs-Exo in the DA neurons of the midbrain at each time point using immunofluorescence. The number of mice at each time point was three. Scale bars, upper, 20 µm; lower, 5 µm. **b** Schematic of in vivo experimental protocol. **c** Open field experiment. **d**–**f** Representative images and quantification of IHC of TH-positive neurons in the striatum and SNpc of the control, T-MSCs-Exo, MPTP, and MPTP + T-MSCs-Exo groups (n = 3 per group). Scale bars, 2.5 mm for images in striatum; 625, 200, and 100 µm for the series of images in SNpc. **g**, **h** Immunofluorescence staining and quantification of TH expression in MPTP-induced PD mice after treatment with T-MSCs-Exo (n = 3 per group). Scale bars, upper, 100 µm; lower, 50 µm. **i**, **j** Western blotting analysis showed the TH expression levels in the SN of the control, T-MSCs-Exo, MPTP, and MPTP + T-MSCs-Exo groups (n = 3 per group). The results are shown as mean ± SD. One-way ANOVA was used to analyze the data. *p < 0.05, **p < 0.01

The BBB hinders effective brain-targeted drug delivery and likely undermines strategies to exploit systemic administration for PD. Therefore, we explored whether T-MSCs-Exo could overcome this limitation. T-MSCs-Exo displayed high efficiency in brain-targeted delivery as assessed by fluorescence imaging of the brain in vitro and in vivo (Additional file 1: Fig. S5a, b). To determine the cell-specific locations of T-MSCs-Exo, we performed immunofluorescence staining with several cell-specific markers: NeuN for neurons, GFAP for astrocytes, and Iba1 for microglia. Immunofluorescence images revealed that PKH26-labelled T-MSCs-Exo were predominantly located in neurons throughout the brain (Additional file 1: Fig. S5c–e). In addition, to further assess the SNpc targeting ability of T-MSCs-Exo, we injected PKH26-labeled T-MSCs-Exo into mice via the tail vein at 0, 2, 6, 8, 10, 12, and 48 h. Immunofluorescence staining was performed for microscopic examination. The results showed that the residence time of T-MSCs-Exo in the mice was prolonged and the uptake by DA neurons in the SNpc was gradually increased (Fig. 3a). These data further confirmed that T-MSCs-Exo not only crossed the BBB, but were also effectively taken up by DA neurons in the SNpc, providing a powerful experimental foundation for subsequent studies of the therapeutic effects of T-MSCs-Exo.

To assess whether T-MSCs-Exo could also produce similar therapeutic effects to T-MSCs on PD, we constructed a chronic PD model by intraperitoneal injection of MPTP (25 mg/kg) into C57BL/6 mice. Different quantities of T-MSCs-Exo were administered in the tail vein on days 3, 17, and 31 after the start of modeling (Fig. 3b). The behavioral tests showed that compared to the MPTP + T-MSCs-Exo (2.44 × 10^10^ particles/mL) group or the MPTP + T-MSCs-Exo (1.22 × 10^11^ particles/mL) group, the MPTP + T-MSCs-Exo (7.32 × 10^10^ particles/mL) group was better able to improve the motor function deficits in the open field test (Fig. 3c; Additional file 1: Fig. S6a–d), rotarod test (Additional file 1: Fig. S6e), hang test (Additional file 1: Fig. S6f), and pole test (Additional file 1: Fig. S6g). Additionally, the protein findings were consistent with this observation (Additional file 1: Fig. S6h, i). In the present study, 7.32 × 10^10^ particles/mL T-MSCs-Exo was selected for further validation by IHC, immunofluorescence and western blotting. The IHC and fluorescence results showed that T-MSCs-Exo-treated PD mice had greater TH density and quantities of TH-positive neurons in the striatum and SNpc than the MPTP group (Fig. 3d–h). Interestingly, the results were also confirmed by western blotting (Fig. 3i, j). These results reveal that T-MSCs-Exo effectively improves behavioral performance and inhibits DA neuron loss in the MPTP-induced PD mice.

### miR-100-5p exerts protective effect on MPP^+^-induced MN9D cells by directly targeting the 3′ UTR of *NOX4*

**Fig. 4:**
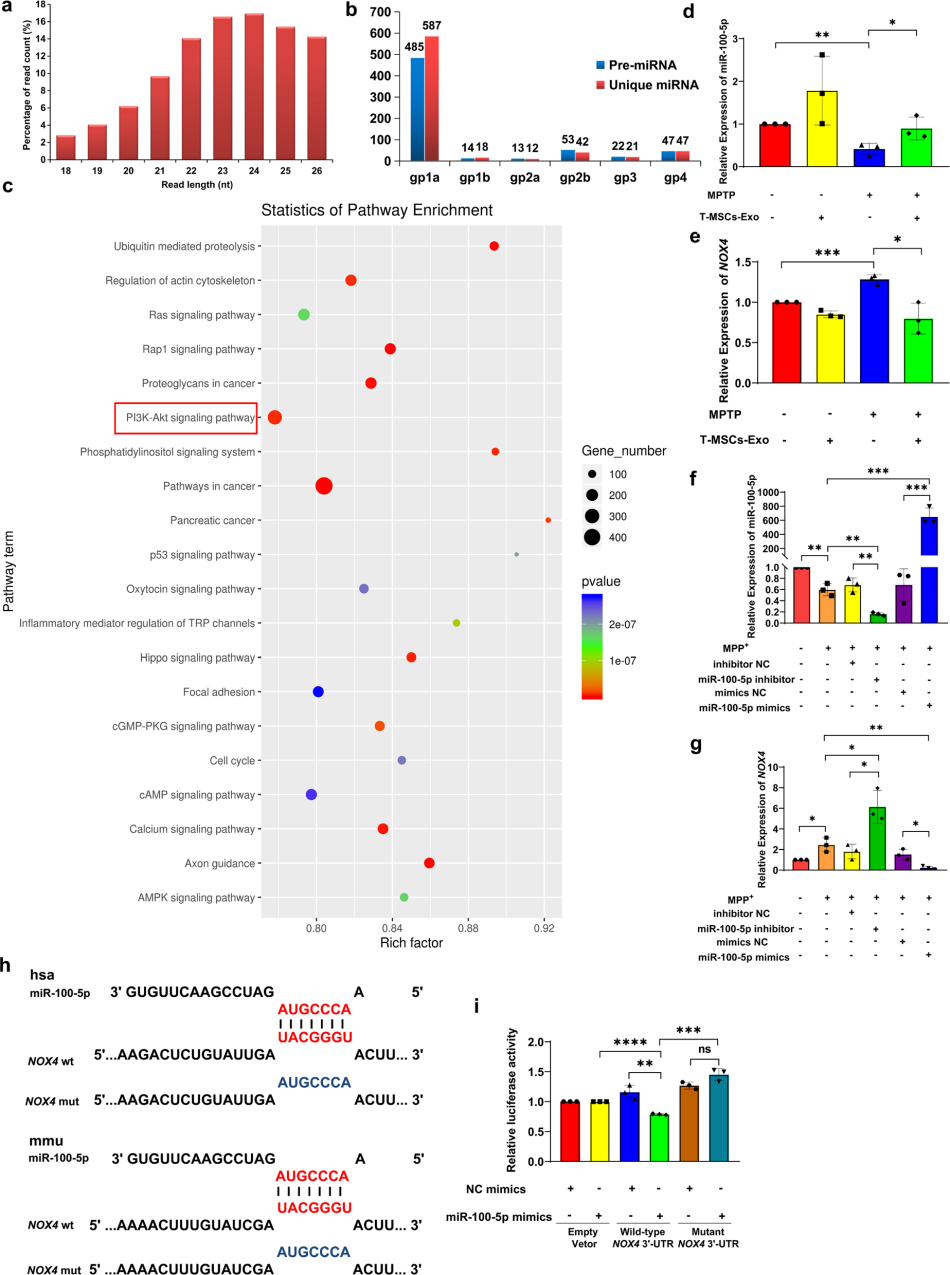
Sequencing analysis of T-MSCs-Exo miRNAs, and miR-100-5p directly targets the 3' UTR *NOX4*. **a** Size distribution of miRNA reads was between 18 and 26 nt with a peak of 23 nt. **b** Summary of known and predicted miRNAs. **c** KEGG pathway analysis. **d**, **e** qRT-PCR assay of miR-100-5p and *NOX4* expression after T-MSCs-Exo treatment of control or MPTP-induced PD mice. **f**, **g** qRT-PCR assay of miR-100-5p and *NOX4* expression in control or MPP^+^-induced MN9D cells after miR-100-5p inhibitor/mimic transfection. **h** Wild-type and mutated-type binding sites between miR-100-5p and *NOX4.* **i** Dual luciferase reporter assay of MN9D cells in the presence of indicated treatments. Each experiment was independently repeated three times. The results are shown as mean ± SD. One-way ANOVA was used to analyze the data. *p < 0.05, **p < 0.01, ***p < 0.001, ****p < 0.0001, and ns: no significant

Exosomes contain proteins, mRNAs, miRNAs and lipids, thereby providing a method for intercellular material transport and information transfer [31]. Next, miRNA sequencing was performed to explore how T-MSCs-Exo ameliorate DA neuron degeneration in PD. After filtering the raw data, the size distribution of miRNA reads was between 18 and 26 nt with a peak of 23 nt (Fig. 4a). Additionally, 485 pre-miRNAs were detected in gp1a (known miRNAs), which accounted for 76.5% of the 634 pre-miRNAs in the miRBase (Fig. 4b). The expressed top 10 miRNAs (including miR-100-5p related to PD) are listed in Table 1 according to the value of norm data. Gene Ontology (GO) classification and Kyoto Encyclopedia of Genes and Genomes (KEGG) pathway analysis revealed the involvement of the PI3K-Akt and AMPK signaling pathways, which have been reported be related to neurodegeneration, especially in PD (Fig. 4c; Additional file 1: Fig. S7).

**Table 1:** Abundant miRNAs (top 10) expressed in T-MSCs-Exo derived from T-MSCs

miR_name	miR_seq	Len	GenomeID	Group
hsa-miR-100-5p	AACCCGTAGATCCGAACTTGT	21	chr11	gp1a
hsa-miR-21-5p	TAGCTTATCAGACTGATGTTGA	22	chr17	gp1a
hsa-miR-320a-3p	AAAAGCTGGGTTGAGAGGGCGA	22	chr8	gp1a
hsa-miR-221-3p	AGCTACATTGTCTGCTGGGTTTC	23	X	gp1a
hsa-miR-146a-5p	TGAGAACTGAATTCCATGGGTT	22	chr5	gp1a
hsa-miR-335-5p	TCAAGAGCAATAACGAAAAATGT	23	chr7	gp1a
hsa-miR-423-5p	TGAGGGGCAGAGAGCGAGACTTT	23	chr17	gp1a
hsa-miR-191-5p	CAACGGAATCCCAAAAGCAGCTG	23	chr3	gp1a
hsa-miR-92a-3p	TATTGCACTTGTCCCGGCCTGT	22	chr13	gp1a
hsa-miR-30e-5p	TGTAAACATCCTTGACTGGAAGCT	24	chr1	gp1a

Bioinformatics analysis showed that the NADPH oxidase 4 (Nox4) gene *NOX4* may be a target for miR-100-5p, which acts on *NOX4* to regulate Nox4 expression. Meanwhile, GO analysis predicted that miR-100-5p was involved in the regulation of oxidative stress in cells (Additional file 1: Fig. S8). Therefore, we validated the expression of *NOX4* and miR-100-5p in PD mice by qRT-PCR. Compared to the MPTP group, miR-100-5p expression was upregulated in the MPTP + T-MSCs-Exo group, whereas the mRNA expression of *NOX4* was downregulated, which is consistent with previous studies [32] (Fig. 4d, e). Meanwhile, we further found that miR-100-5p inhibitor treatment increased the mRNA expression of *NOX4* compared to that of a negative control (NC) inhibitor. Additionally, miR-100-5p mimics significantly reduced the *NOX4* levels (Fig. 4f, g). For this reason, to confirm whether miR-100-5p targets this gene directly, we cloned the 3′ UTRs of the putative targets into a luciferase reporter construct. Reporter assays revealed that miR-100-5p significantly repressed the *NOX4* 3′ UTR (Fig. 4h, i). Collectively, these results indicate that T-MSCs-Exo miR-100-5p downregulated *NOX4* expression, by directly targeting the 3′ UTR of *NOX4* to ameliorate DA neuron degeneration in PD.

### T-MSCs alleviate oxidative stress injury in PD models through the Keap1-Nrf2-SOD pathway

**Fig. 5:**
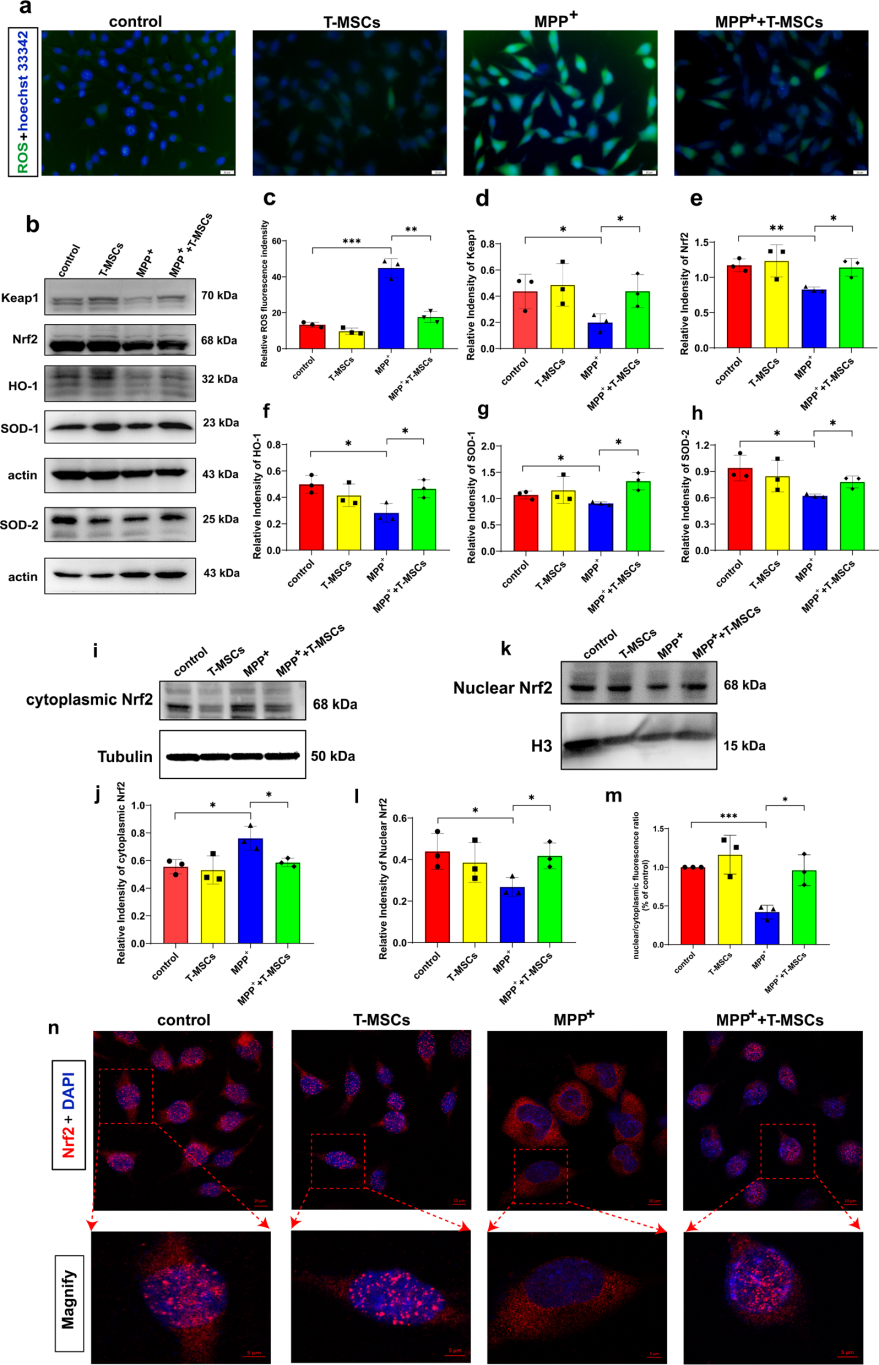
T-MSCs alleviate oxidative stress injury in MPP^+^-induced MN9D cells through the Keap1-Nrf2-SOD pathway. **a** Representative graphs of ROS generation after T-MSCs treatment of control or MPP^+^-induced MN9D cells. Scale bars, 20 µm. **b,** **d**–**h** Western blotting analysis of Keap1, Nrf2, HO-1, SOD-1, and SOD-2 expression levels after T-MSCs treatment of control or MPP^+^-induced MN9D cells. **c** Quantification of the relative fluorescence intensity of ROS levels. **i**–**l** Representative blots and quantification showed the levels of cytoplasmic Nrf2 and Nuclear Nrf2 in MN9D cells. **m**, **n** Immunofluorescence staining and quantification of Nrf2 in the control, T-MSCs, MPP^+^, and MPP^+^ + T-MSCs groups. Scale bars, upper, 10 µm; lower, 5 µm. Each experiment was independently repeated three times. The results are shown as mean ± SD. One-way ANOVA was used to analyze the data. *p < 0.05, **p < 0.01, ***p < 0.001

Multiple factors, including oxidative stress, may play roles in the etiology of PD. The production of ROS is an important manifestation of mitochondrial oxidative stress [33]. Therefore, we labeled MN9D cells with 2', 7'-dichlorodihydrofluorescein diacetate (DCFH)-DA probe and detected ROS levels. The results indicate that ROS levels were significantly enhanced in MPP^+^-induced MN9D cells compared to the control group, while ROS levels were reduced in the MPP^+^ + T-MSCs group compared to the MPP^+^ group (Fig. 5a, c). These results suggest that T-MSCs can reduce ROS production in MPP^+^-induced MN9D cells.

As the Keap1-Nrf2-SOD pathway plays a fundamental role in neurodegenerative disorders including PD [34, 35], we examined the effect of T-MSCs on the Keap1-Nrf2-SOD pathway in MPP^+^-induced MN9D cells. The results showed that the expression levels of Keap-1, Nrf2, HO-1, SOD-1 and SOD-2 were higher in the MPP^+^ + T-MSCs group than in the MPP^+^ group (Fig. 5b, d–h). Additionally, when excessive prooxidant stimulation causes the dissociation of Nrf2 from Keap1, Nrf2 translocates to the nucleus to activate the expression of antioxidant enzymes [36]. For this reason, we used western blotting and immunofluorescence staining to detect the nuclear translocation of Nrf2. The data displayed increased expression of Nrf2 in the nucleus in the MPP^+^ + T-MSCs group compared to the MPP^+^ group, with similar effects demonstrated in fluorescence (Fig. 5i–n). To further explore the mechanism of the Keap1-Nrf2-SOD pathway involved in the protection of DA neurons in T-MSCs, we used ML385, an inhibitor of Nrf2. Western blotting assays showed that Keap-1, Nrf2, HO-1, SOD-1, and SOD-2 expression levels were significantly decreased in the MPP^+^ + T-MSCs + ML385 group compared to the MPP^+^ + T-MSCs group. Although these markers were downregulated after MPP^+^ induction, no significant differences were observed in the MPP^+^ + ML385 group compared to the MPP^+^group (Additional file 1: Fig. S9). These data demonstrate that T-MSCs intervention can upregulate the expression of the antioxidant enzymes SOD-1 and SOD-2 through the Keap1-Nrf2-SOD pathway to enhance the antioxidant capacity of MPP^+^-induced MN9D cells.

**Fig. 6:**
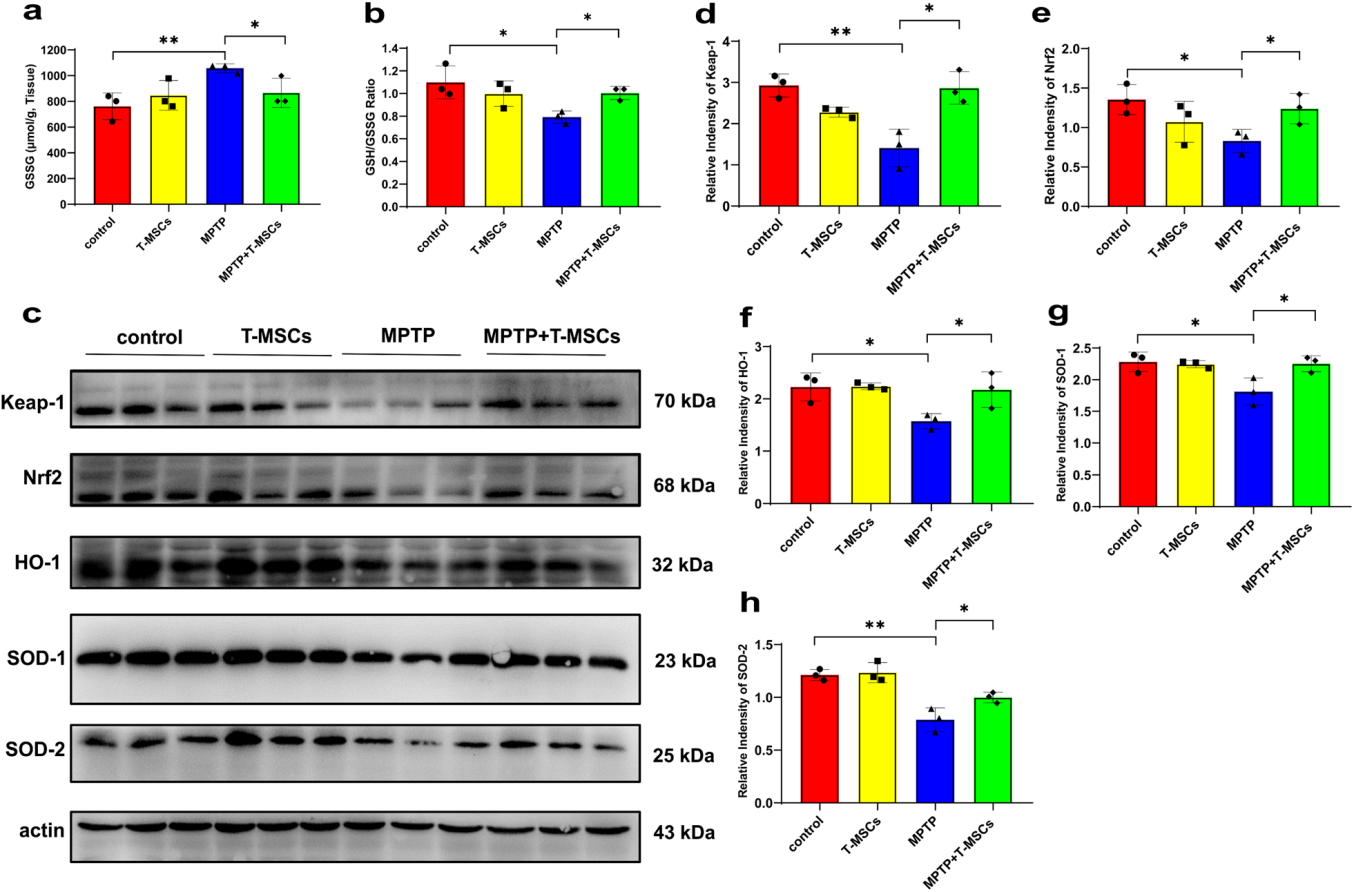
T-MSCs resist oxidative stress injury in PD mice through Keap1-Nrf2-SOD. **a**, **b** The content of GSSG and relative GSH/GSSG ratio after T-MSCs treatment of control or MPTP-induced PD mouse model (n = 3 per group). **c**–**h** Western blotting analysis of Keap1, Nrf2, HO-1, SOD-1, and SOD-2 expression levels after T-MSCs treatment of control or MPTP-induced PD mouse model (n = 3 per group). The results are shown as mean ± SD. One-way ANOVA was used to analyze the data. *p < 0.05, **p < 0.01

In vivo, reduced glutathione (GSH) is the major intracellular antioxidant peptide and enhances antioxidant defense [37]. The GSH/oxidized glutathione (GSSG) ratio is often used as an indicator of cytotoxicity. Hence, we evaluated the GSSG and GSH/GSSG ratio in PD mice. The results showed that PD mice treated with T-MSCs had reduced GSSG levels and increased GSH/GSSG ratios (Fig. 6a, b). Next, we analyzed whether a similar antioxidant effect to T-MSCs was present in PD mice. The results were consistent with those of in vitro experiments. The expression levels of Keap-1, Nrf2, HO-1, SOD-1 and SOD-2 were increased after T-MSCs treatment of PD mice (Fig. 6c–h). These results demonstrate that T-MSCs enhance the antioxidant capacity of PD mice by activating the Keap1-Nrf2-SOD pathway.

Collectively, these results suggest that T-MSCs are involved in decreasing the intracellular ROS levels, and improving the concentration of reduced glutathione (or GSH/GSSG ratio) in the brain of PD mice to a certain extent. Meanwhile, through the Keap1-Nrf2-SOD pathway, T-MSCs can upregulate the expression of the antioxidant enzymes SOD-1 and SOD-2, and increase nuclear Nrf2 expression, thereby further enhancing the antioxidant capacity in PD.

### T-MSCs-Exo equally attenuate oxidative stress damage via activation of the Keap1-Nrf2-SOD pathway in PD, similar to T-MSCs

**Fig. 7:**
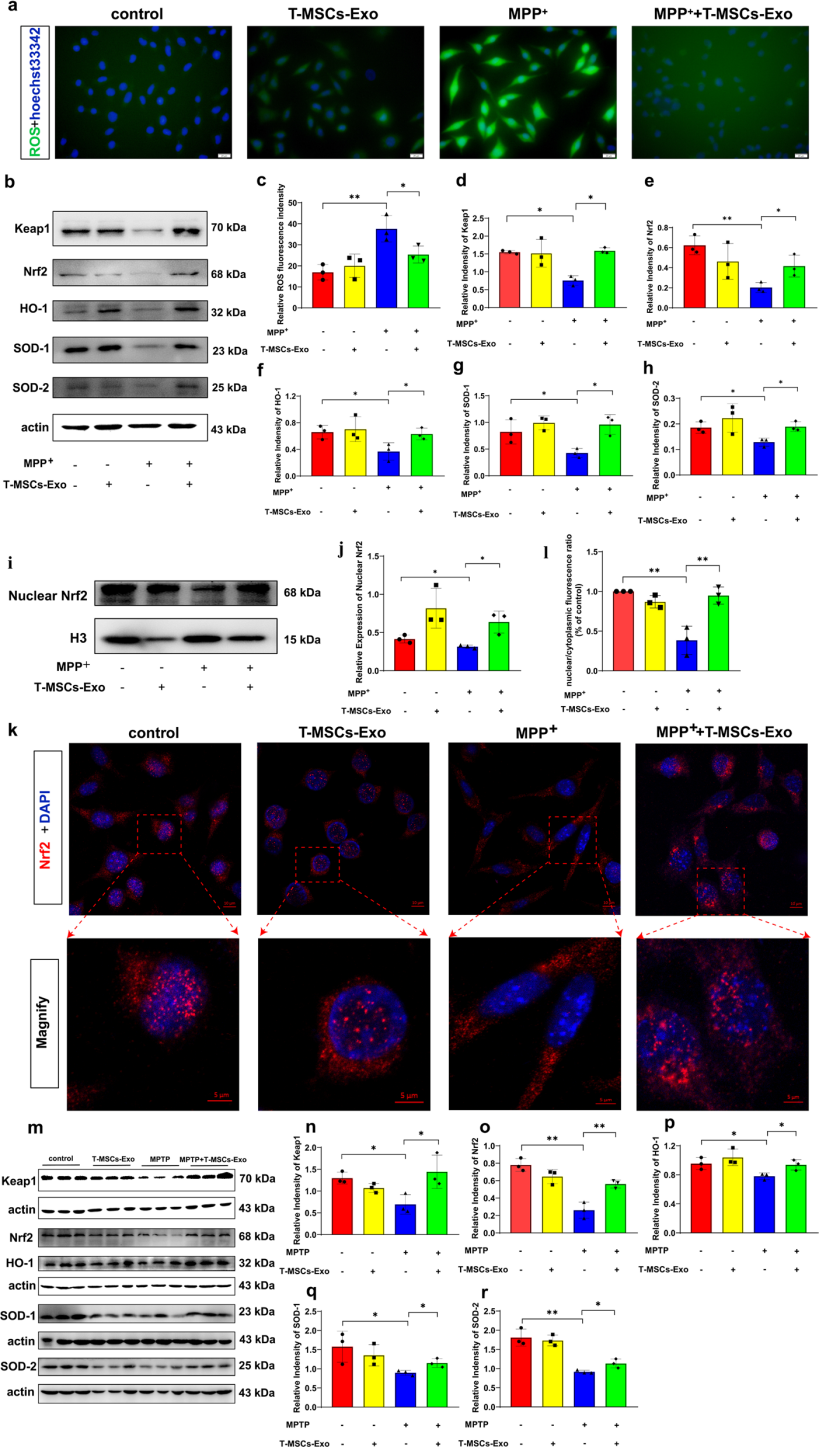
T-MSCs-Exo alleviate oxidative stress injury in PD models through Keap1-Nrf2-SOD. **a** Representative graphs of ROS generation after T-MSCs-Exo treatment of control or MPP^+^-induced MN9D cells. Scale bars, 20 µm. **b**, **d–h** Western blotting analysis of Keap1, Nrf2, HO-1, SOD-1, and SOD-2 expression levels after T-MSCs-Exo treatment of control or MPP^+^-induced MN9D cells. **c** Quantification of the relative fluorescence intensity of ROS levels. **i**, **j** Representative blots and quantification showed the levels of nuclear Nrf2 in MN9D cells. **k**, **l** Immunofluorescence staining and quantification of Nrf2 in the control, T-MSCs-Exo, MPP^+^, and MPP^+^ + T-MSCs-Exo groups. **m-r** Western blotting analysis of Keap1, Nrf2, HO-1, SOD-1, and SOD-2 expression levels after T-MSCs-Exo treatment of control or MPTP-induced PD mouse model (n = 3 per group). Scale bars, upper, 10 µm; lower, 5 µm. Each experiment was independently repeated three times. The results are shown as mean ± SD. One-way ANOVA was used to analyze the data. *p < 0.05, **p < 0.01

Previously, we demonstrated that T-MSCs reduce ROS production in MPP^+^-induced MN9D cells. Howerver, it is unclear whether T-MSCs-Exo, a secreted product of T-MSCs, also exert a similar effect. The results showed that ROS levels were significantly enhanced in MPP^+^-induced MN9D cells compared to the control group, while ROS levels were reduced in the MPP^+^ + T-MSCs-Exo group compared to the MPP^+^ group (Fig. 7a, c). These results suggest that T-MSCs-Exo can reduce ROS production in MPP^+^-induced MN9D cells.

Next, we detected whether T-MSCs-Exo could also be involved in upregulating the SOD-1 and SOD-2 expression via the Keap1-Nrf2-SOD pathway in vitro. The results indicated that the expression levels of Keap-1, Nrf2, HO-1, SOD-1, and SOD-2 were increased in the MPP^+^ + T-MSCs-Exo group compared to the MPP^+^ group (Fig. 7b, d–h). At the same time, we examined the effect of T-MSCs-Exo on nuclear Nrf2 expression. The data showed increased expression of Nrf2 in the nucleus in the MPP^+^ + T-MSCs-Exo group compared to the MPP^+^ group, with similar effects demonstrated by fluorescence staining (Fig. 7i–l).

To determine the role of T-MSCs-Exo in the regulation of neuroprotection in MN9D cells exposed to MPP^+^, we used an inhibitor of exosome biogenesis/release, GW4869, to pretreat T-MSCs, thereby inhibiting the release of exosomes from T-MSCs. The results showed that the expression levels of TH, Keap-1, Nrf2, HO-1, SOD-1 and SOD-2 were significantly decreased in the MPP^+^ + T-MSCs-Exo + GW4869 group compared to the MPP^+^ + T-MSCs-Exo group (Additional file 1: Fig. S10a–i). In addition, we used ML385, an inhibitor of Nrf2, to further explore the mechanism of the Keap1-Nrf2-SOD pathway involved in the protection of DA neurons in T-MSCs. Western blotting assays showed that Keap-1, Nrf2, HO-1, SOD-1 and SOD-2 expression levels were significantly decreased in the MPP^+^ + T-MSCs-Exo + ML385 group compared to the MPP^+^ + T-MSCs-Exo group (Additional file 1: Fig. S10j–o). These data reveal that T-MSCs are most likely involved in the upregulation of SOD-1 and SOD-2 expression via the secretion of T-MSCs-Exo, thereby activating the Keap1-Nrf2-SOD pathway to enhance the antioxidant capacity of MN9D cells.

Next, we analyzed whether a similar antioxidant effect of T-MSCs-Exo was present in PD mice. The results were consistent with our earlier in vitro results. The expression levels of Keap-1, Nrf2, HO-1, SOD-1 and SOD-2 were increased after T-MSCs-Exo treatment of PD mice (Fig. 7m–r). Taken together, T-MSCs-Exo, a product secreted by T-MSCs, are not only involved in the protective effects of PD, but also exert antioxidant effects on PD by activating the Keap1-Nrf2-SOD pathway.

### Overexpression of miR-100-5p ameliorates oxidative stress damage in PD via the Nox4-ROS-Nrf2 axis

**Fig. 8:**
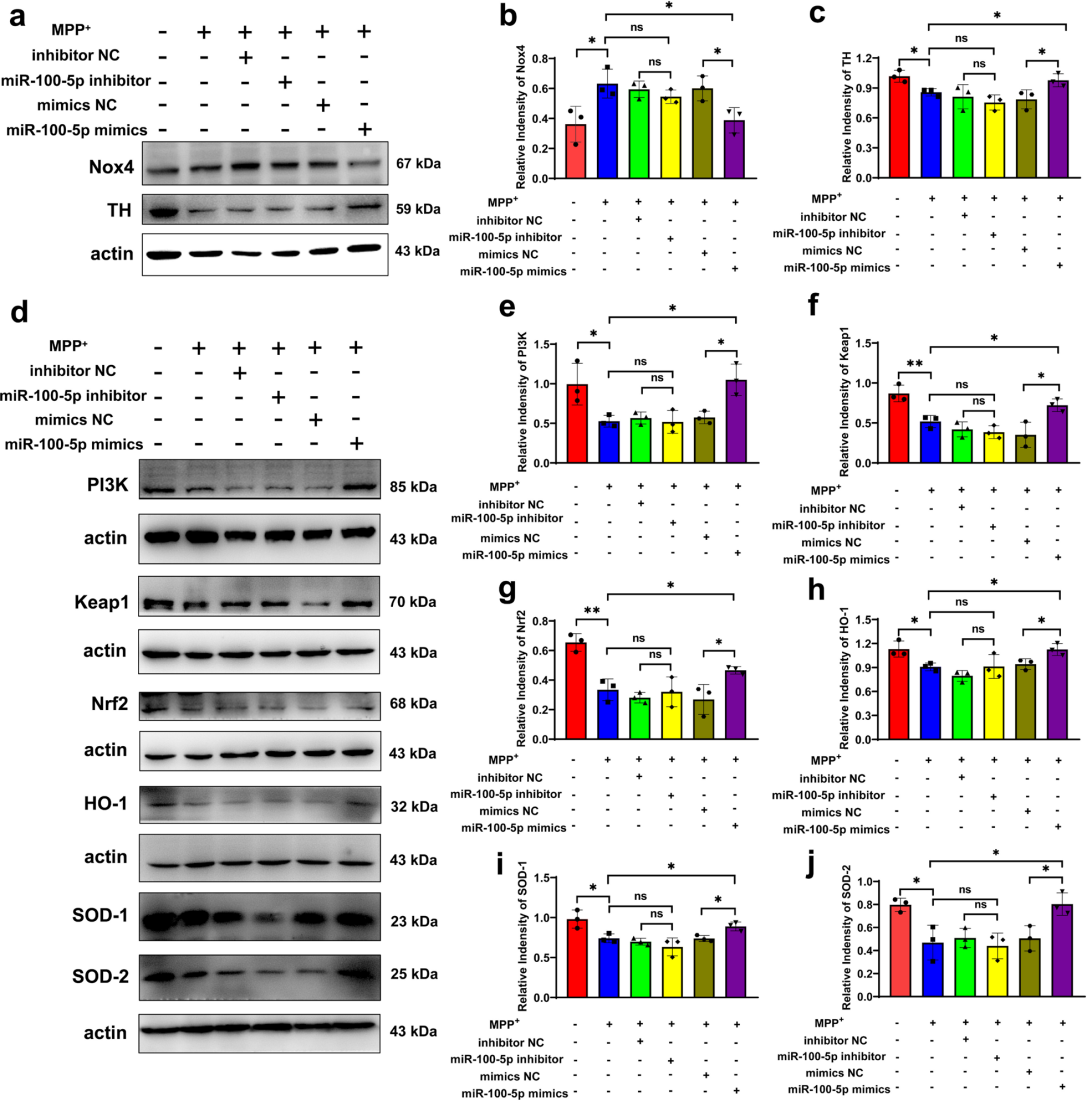
miR-100-5p relieves oxidative stress injury in MPP^+^-induced MN9D cells via the Nox4-ROS-Nrf2 axis. **a**–**c** Representative blots and quantification showed the levels of Nox4 and TH in control and MPP^+^-induced MN9D cells after miR-100-5p inhibitor/mimics transfection. **d**–**j** Western blotting analysis of PI3K, Keap1, Nrf2, HO-1, SOD-1, and SOD-2 in control or MPP^+^-induced MN9D cells after miR-100-5p inhibitor/mimic transfection. Each experiment was independently repeated three times. The results are shown as mean ± SD. One-way ANOVA was used to analyze the data. *p < 0.05, **p < 0.01, *ns* no significant

To shed light on the functionality of miR-100-5p in MPP^+^-induced pathologies, we evaluated the expression of Nox4 in MN9D cells transfected with miR-100-5p inhibitor, inhibitor NC, mimics, and mimics NC. Overexpression of miR-100-5p dramatically reduced the protein levels of Nox4 and increased the TH expression in MPP^+^-induced MN9D cells (Fig. 8a–c). Unfortunately, the miR-100-5p inhibitor did not upregulate the effect on the Nox4 protein level compared to the MPP^+^ + inhibitor NC group, but did not further exacerbate to the DA neuron damage. (Fig. 8a–c). These results indicate that overexpression of miR-100-5p significantly inhibited MPP^+^-mediated Nox4 protein expression, thereby enhancing the protective effect against the PD cell model.

**Fig. 9:**
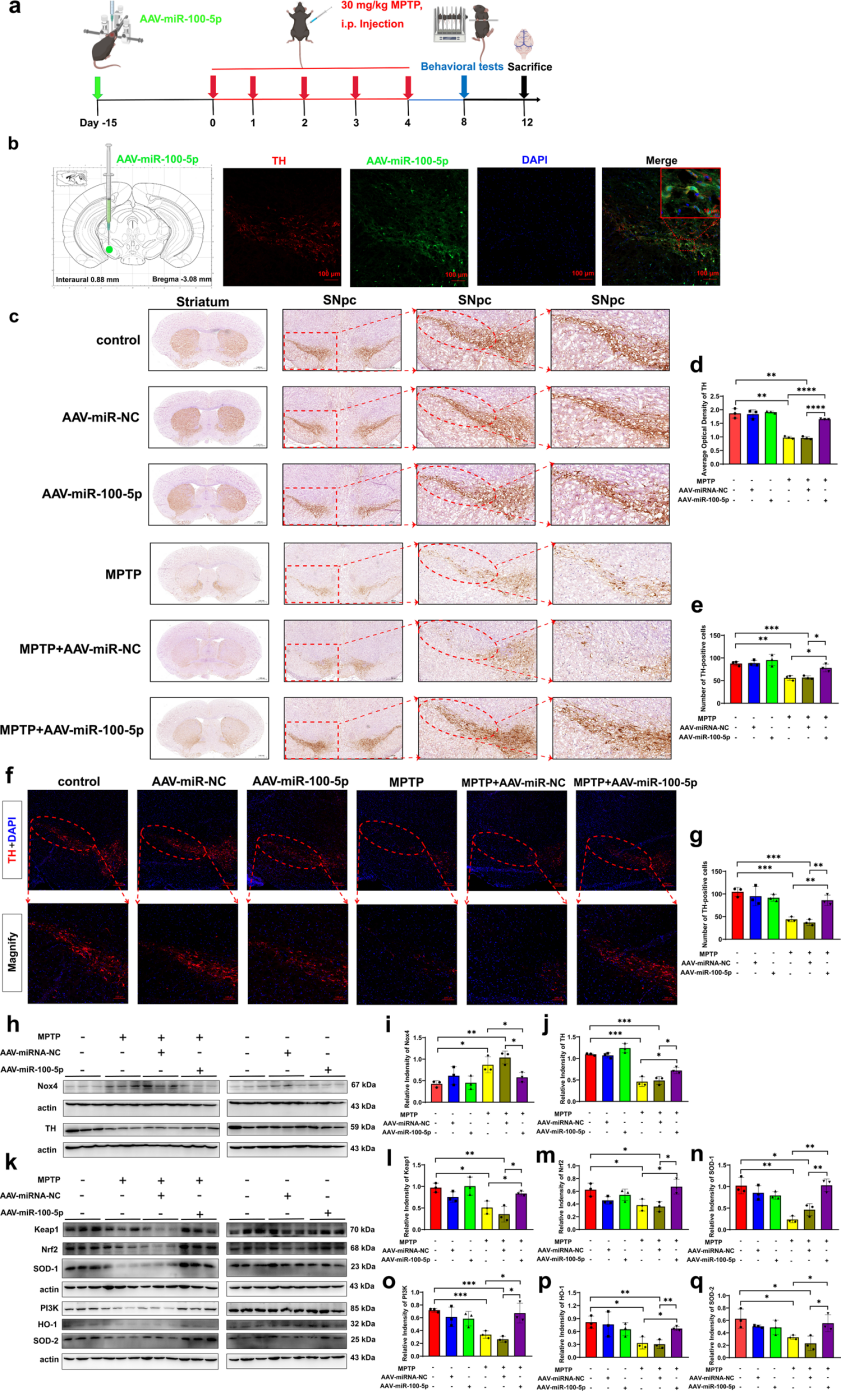
miR-100-5p relieves oxidative stress injury in MPTP-induced PD mice via the Nox4-ROS-Nrf2 axis. **a** Design and timeline of the animal experimental procedure. **b** Representational diagram of AAV injection site in mice and the co-labeling of TH with miR-100-5p showed that miR-100-5p was mainly expressed within the SNpc DA neurons. Scale bars, 100 µm; upper right, 10 µm. **c**–**e** Representative images and quantification of IHC of TH-positive neurons in the striatum and SNpc of the control, AAV-miR-NC, AAV-miR-100-5p, MPTP, MPTP + AAV-miR-NC, and MPTP + AAV-miR-100-5p groups (n = 3 per group). Scale bars, 1.000 mm for images in striatum; 500, 200, and 100 µm for the series of images in SNpc.** f**–**g** Immunofluorescence staining and quantification of TH expression in MPTP-stimulated PD mice after treatment with AAV-miR-NC or AAV-miR-100-5p (n = 3 per group). Scale bars, upper, 200 µm; lower, 100 µm. **h**–**j** Western blotting analysis showed the Nox4 and TH expression levels in the SN of the six groups (n = 3 per group). **k**–**q** Western blotting analysis showed the expression levels of PI3K, Keap1, Nrf2, HO-1, SOD-1, and SOD-2 in the SN of the six groups (n = 3 per group). The results are shown as mean ± SD. One-way ANOVA was used to analyze the data. *p < 0.05, **p < 0.01, ***p < 0.001, and ****p < 0.0001

Our study proved that *NOX4* is a direct target of miR-100-5p. Nox4 is an isoform of NADPH oxidase, which produces more ROS than other Nox proteins. The elevated ROS level leads to oxidative stress, which is associated with inflammation and degenerative diseases (including PD) [38, 39]. Therefore, we speculated that miR-100-5p could play a crucial role via the Nox4-ROS-Nrf2 axis in PD. In vitro, we transfected miR-100-5p inhibitor, inhibitor NC, mimics, and mimics NC into MPP^+^-induced MN9D cells. Western blotting analysis showed that the expression levels of PI3K, Keap-1, Nrf2, HO-1, SOD-1 and SOD-2 were increased in the MPP^+^ + miR-100-5p mimics group compared to the MPP^+^ + mimics NC group (Fig. 8d–j). Next, we used MPTP to create a PD mouse model 15 days after stereotaxic injection of AAV-miR-100-5p into the left SNpc (Fig. 9a, b). The motor function in mice and the changes in the expression levels of various molecules in the Nox4-ROS-Nrf2 pathway were assessed. Behavioral studies showed that compared to the MPTP + AAV-miRNA-NC group, the MPTP + AAV-miR-100-5p group was better able to improve the motor function deficits in the open field test (Additional file 1: Fig. S11a–d), rotarod test (Additional file 1: Fig. S11e), hang test (Additional file 1: Fig. S11f), and pole test (Additional file 1: Fig. S11g). In the present study, we further verified this phenomenon by IHC and immunofluorescence. The IHC and fluorescence results showed that overexpression of miR-100-5p in PD mice was associated with greater TH density and quantities of TH-positive neurons in the striatum and SNpc compared to the MPTP + AAV-miRNA-NC group (Fig. 9c–g). Interestingly, the results were also confirmed by western blotting. Compared to the MPTP + AAV-miRNA-NC group, the MPTP + AAV-miR-100-5p group could better suppress the expression of Nox4 while further restoring the TH expression level (Fig. 9h–j). Furthermore, we validated the protein expression changes of various molecules in the Nox4-ROS-Nrf2 axis. The western blotting results were similar to those of the in vitro experiments. The expression levels of PI3K, Keap-1, Nrf2, HO-1, SOD-1, and SOD-2 were increased in the MPTP + AAV-miR-100-5p mimics group compared to the MPTP + AAV-miRNA-NC group (Fig. 9k–q). The inverse-correlated expression profile of miR-100-5p and *NOX4* indicates that miRNA-regulated Nox4 pathway may play a fundamental role in PD progression.

Overall, the presented results demonstrate the potential uses of T-MSCs and T-MSCs-Exo for PD. T-MSCs with the ability to differentiate from TB-like cells improved motor deficits in a PD mouse model and increased TH expression in MPP^+^-induced MN9D cells, indicating their neuroprotective effects. Meanwhile, T-MSCs-Exo, isolated from T-MSCs, were shown to be internalized by MN9D cells and enhanced TH expression, suggesting that they play a role in promoting DA neurons. Furthermore, T-MSCs-Exo demonstrated the ability to cross the BBB and selectively target DA neurons in the SNpc, providing a potential delivery system for therapeutic agents. Additionally, miRNA sequencing identified miR-100-5p as an abundant miRNA in T-MSCs-Exo, and subsequent in vitro and in vivo experiments confirmed its protective effect in DA neurons. MiR-100-5p was found to target the 3' untranslated region (UTR) of *NOX4*, leading to the downregulation of Nox4 expression and amelioration of DA neuron degeneration via the miR-100-5p-Nox4-ROS-Nrf2 axis. Together, these findings highlight the potential uses of T-MSCs-Exo as therapeutic strategies for PD through their neuroprotective effects, regulation of oxidative stress, and modulation of specific molecular targets. Further research is warranted to investigate the optimal dosing, treatment duration, and long-term safety of T-MSCs-Exo, as well as their potential synergistic effects with other therapeutic approaches. Additionally, understanding the broader mechanisms underlying their effects and exploring their impact on other aspects of PD pathology would contribute to the development of comprehensive and targeted therapies for PD patients.

## Discussion

PD is a long-term degenerative condition that progresses over time, causing the specific loss of DA neurons and α-syn aggregation in the SNpc [40]. The current treatment of PD is still based on dopamine supplementation and surgical treatment (e.g. DBS) to relieve clinical symptoms [41]. Domestic and foreign studies have shown that MSCs repair damaged tissues in neurodegenerative diseases, exerting therapeutic effects by releasing immunomodulatory and trophic factors, as well as by establishing direct cell-to-cell communication [42]. However, MSCs from different tissue origins exhibit functional heterogeneity, even within the same tissue type. In addition, the cell quality may be impacted by several factors including donor variation, the method of isolation, and the amplification process [43]. MSCs derived from embryonic stem cells exhibit a consistent phenotype, possess enduring immunomodulatory capabilities, and can be easily cultured on a large scale [15]. Meanwhile, they have therapeutic potential for multiple sclerosis or dextran sulfate sodium-induced colitis animal model [16]. One study reported that when human embryonic stem cell-derived mesenchymal stem/stromal cells (hES-MSCs) were in direct contact with brain microvascular endothelial cells (BMECs), TNF-α-induced changes in tight junction proteins, permeability, trans-endothelial resistance, and adhesion molecule expression were reversed, thereby repairing the neuroinflammation-associated BBB damage accompanying experimental autoimmune encephalomyelitis (EAE) [44]. Furthermore, in another study, intravenous infusion of T-MSCs was found to attenuate dextrose sodium sulfate (DSS)-induced colitis in mice by elevating the level of circulating IGF-1 through the IGF1R-PI3K-AKT pathway, thereby preserving the integrity of the colonic epithelial cells and facilitating their restoration and rejuvenation [45]. Additionally, human ESC-derived MSCs (EMSC) promoted angiogenesis and adipocyte survival through adipocyte reunification, CCL2 signaling activation, and macrophage recruitment [46]. Based on the abovementioned research, we used phenotypically uniform T-MSCs from commercial ESI-053 embryonic stem cells, which are capable of stable production, and their exosomes (T-MSCs-Exo) to explore the molecular mechanisms involved in DA neuron protection in PD models. The initial findings of our study showed that compared to the MPTP group, the MPTP + T-MSCs group improved the motor function deficits in the open field test, rotarod test, hang test, and pole test (Additional file 1: Fig. S3a–g). This suggests that a PD-related motor pattern is related with the capacity for motor improvement after T-MSCs treatment, in line with our expected assumptions.

As is well-known, the most consistent pathological finding in PD is the loss of SNpc DA neurons and subsequent depletion of striatal dopamine [47]. TH, the rate-limiting enzyme in the biosynthesis of dopamine, is considered a marker of DA neurons. Western blotting and immunohistochemical results in vitro and in vivo confirm that T-MSCs have a protective effect against DA neuron damage from PD (Fig. 1; Additional file 1: Fig. S2). In addition, we also found that treatment with T-MSCs reduced α-syn aggregation in PD mice (Additional file 1: Fig. S3h–i). This will provide the basis for further experimental explorations on the underlying mechanism.

To further investigate the mechanism by which T-MSCs have a protective effect against PD, we used polyethylene glycol precipitation to extract the contents from the supernatant of T-MSCs and purify them for identification (Additional file 1: Fig. S4), which was eventually verified as exosomes, in line with the criteria for exosomes reported in the literature [20]. Furthermore, exosomes are lipid-bound nanoscale vesicles that freely cross the BBB, reducing inflammation in the brain and ultimately helping to repair damaged neurons [2, 21]. In our study, T-MSCs-Exo, generated from T-MSCs, have demonstrated internalization by MN9D cells, further augmenting TH expression, and thereby underscoring their potential role in fostering the survival of DA neurons. Furthermore, T-MSCs-Exo exhibited the capability to cross the BBB and selectively target DA neurons in the SNpc, thereby elucidating a plausible system for the delivery of therapeutic agents.

Emerging evidence has suggested that exosomes, which contain miRNAs, mRNAs, and proteins, play important roles in cell-to-cell communication as natural carriers [30]. As important components of the exosomes, miRNAs regulate a variety of injuries and diseases, including PD. Some miRNAs found in the exosomes, may have therapeutic effects due to their role in the regulation of matrix synthesis. An example is when exosomes obtained from adipose-derived mesenchymal stem cells that were modified with miR-188-3p inhibited autophagy and pyroptosis in PD mice and MN9D cells by specifically targeting CDK5 and NLRP3 [23]. Furthermore, researchers demonstrated enhanced insulin sensitivity in elderly mice by reducing the expression of miR-29b-3p in exosomes derived from BM-MSCs using a nanocomplex delivery system facilitated by aptamers. This indicates that targeting exosomal miR-29b-3p could be a promising approach for addressing insulin resistance related to aging [48]. In this study, we confirmed the presence of miRNAs in T-MSCs-Exo isolated from the culture supernatant of T-MSCs by miRNA sequencing. Additionally, 485 pre-miRNAs were detected in gp1a (known miRNAs), which accounted for 76.5% of the 634 pre-miRNAs in the miRBase. Moreover, we screened the top 10 miRNAs (including miR-100-5p) with a high expression level (Table 1).

According to a previous report, the researchers analyzed the substantia nigra from postmortem PD midbrain tissues through the GEO database and screened 15 miRNAs that were significantly associated with PD, including miR-100-5p [49]. However, the specific role of miR-100-5p in PD has not been reported and requires further in-depth study. With the use of bioinformatics analysis, KEGG pathway analysis revealed that exosomal miR-100-5p was associated with the PI3K-Akt signaling pathways, which have been implicated in PD (Fig. 4c; Additional file 1: Fig. S7). Meanwhile, GO analysis predicted that miR-100-5p was involved in the regulation of oxidative stress in cells (Additional file 1: Fig. S8). Based on this, it is currently hypothesized that miR-100-5p may be involved in oxidative stress regulation by targeting *NOX4* to regulate Nox4 expression, so miR-100-5p was preferentially selected for the follow-up experiments. In this study, qRT-PCR analysis validated that miR-100-5p inhibitor treatment increased the expression of *NOX4* compared to that of an NC inhibitor. The miR-100-5p mimics significantly reduced the *NOX4* mRNA level. Furthermore, *NOX4* was validated as a target of miR-100-5p through the dual luciferase reporter assay. Together, the study demonstrates that miR-100-5p downregulates Nox4 expression by directly targeting the 3′ UTR of *NOX4* to improve DA neuron degeneration in PD.

MSCs exert antioxidant effects in many diseases, and the main mechanisms include scavenging of free radicals, promotion of endogenous antioxidant defense, immunomodulation through inhibition of ROS, and alteration of mitochondrial bioenergetics [50–53]. According to the study conducted by Li and colleagues [54], it was discovered that MSCs have the ability to relieve mitochondrial dysfunction in the airways caused by oxidative stress. Meanwhile, this view was subsequently confirmed by Kizilay et al. [55]. Moreover, such a notion was validated by our subsequent mechanism research. T-MSCs are involved in decreasing intracellular ROS levels, as well as increasing the GSH/GSSG ratio in the brain of PD mice to a certain extent. Furthermore, the Keap1-Nrf2-SOD pathway showed that T-MSCs can upregulate the expression of the antioxidant enzymes SOD-1 and SOD-2, and increase nuclear Nrf2 expression, further enhancing the antioxidant ability of PD. In addition, through in vivo and ex vivo experiments, we further confirmed that T-MSCs-Exo, as secretory products of T-MSCs, also play a role in protection against PD via the Keap1-Nrf2-SOD pathway, which provides sufficient experimental data for subsequent in-depth studies.

As one of the isoforms of NADPH oxidase, Nox4 produces large amounts of ROS, leading to oxidative stress, which is linked to numerous neurodegenerative diseases [39]. One study reported that MSC-derived extracellular vesicle (EV)-encapsulated miR-181a-2-3p downregulated EGR1, thus suppressing oxidative stress via the NOX4/p38 MAPK pathway in both PD models, whether in vivo or ex vivo [5]. Moreover, the recent study have demonstrated that the increase in *NOX4* interacts with the neuroinflammatory mediators (MPO and OPN) to control ferroptosis through astrocytic mitochondrial dysfunction in the hippocampus during PD, as observed when comparing an MPTP-induced PD mouse model with human PD patients [56]. Consistently, we confirmed that *NOX4* was a potential target of miR-100-5p using dual-luciferase reporter assay. This suggests that there is a strong possibility that the Nox4-ROS-Nrf2 axis is associated with PD, but further studies will be required to elucidate the fundamental molecular mechanisms involved. In the present study, we focused on changes in the Nox4 expression level in the PD cell model and found that the expression levels of PI3K, Keap-1, Nrf2, HO-1, SOD-1 and SOD-2 were increased in the MPP^+^ + miR-100-5p mimics group compared to the MPP^+^ + mimics NC group by western blotting analysis. However, the miR-100-5p inhibitor did not aggravate PD-like phenotypes in MPP^+^-induced MN9D cells. As such, we propose that this discrepancy may have been due to ineffective inhibition of miR-100-5p in our present study and/or that our miR-100-5p inhibitor was degraded through enzymolysis. To further explore the role of miR-100-5p in PD mice, we used MPTP to create a PD mouse model 15 days after stereotaxic injection of AAV-miR-100-5p into the left SNpc. According to the data, the excessive expression of miR-100-5p can enhance the motor function impairments and recover the level of TH expression in mice with PD through the Nox4-ROS-Nrf2 axis. Together, the present findings further confirm the important role of miR-100-5p in both in vitro and in vivo PD models.

In this study, we demonstrated that T-MSCs-Exo carrying miR-100-5p cross the BBB to exert neuroprotective effects in PD. Nevertheless, there are several limitations to the clinical applicability of T-MSCs-Exo at this time. It is essential to acknowledge the limitations associated with the animal and cell models, which may not perfectly replicate human physiology due to inherent species differences. Furthermore, while the results emphasize the neuroprotective aspects of the miR-100-5p-enriched T-MSCs-Exo treatment, the study falls short of providing a robust investigation into crucial parameters, such as its optimal dosage, frequency, and long-term safety and effectiveness. In addition, the study predominantly focuses on the Nox4-ROS-Nrf2 axis and the regulation of *NOX4* by miR-100-5p, yet the pathological mechanisms of PD are diverse and complex. Therefore, extensive studies should be implemented in the future.

To address these limitations, it is crucial to conduct preclinical studies that delve deeper into the optimal dosing, treatment duration, and long-term effects of T-MSCs and T-MSCs-Exo using normal rats and mice. Meanwhile, the toxicity, tumorigenicity and pharmacokinetic studies of T-MSCs-Exo in vivo should be used to determine the potential toxic target organs and the degree and reversibility of the toxic response in T-MSCs Exo, thus clarifying the safety parameters for clinical monitoring. Furthermore, well-designed clinical trials of PD patients are necessary to rigorously evaluate the safety and efficacy of T-MSCs and T-MSCs-Exo, as well as the establishment of comprehensive and standardized protocols for their production, characterization, and administration to ensure reproducibility and comparability across studies. Moreover, optimizing the delivery methods of miR-100-5p and developing miRNA mimics with enhanced stability and specificity are critical for the future translation of these interventions into clinical practice. By pursuing this multifaceted approach, we can advance our understanding of T-MSCs, T-MSCs-Exo, and miR-100-5p as potential therapeutic strategies and pave the way for improved treatments for PD patients.

## Conclusions

PD is a growing epidemic for which there are currently no effective disease-modifying drugs [57]. Therefore, the development of effective neuroprotective therapeutic agents to halt or delay disease progression remains a high priority. In this study, our research underscores the therapeutic potential of T-MSCs and T-MSCs-Exo in PD treatment, highlighting their capacity to improve motor deficits and provide neuroprotection by elevating TH expression. Meanwhile, the presented results reveal that miR-100-5p-enriched T-MSCs-Exo protect against the loss of DA neurons, maintain nigro-striatal system function, ameliorate motor deficits, and reduce oxidative stress through the Nox4-ROS-Nrf2 axis, suggesting broad neuroprotective properties of T-MSCs-Exo. Despite promising results, further studies should address issues such as species variability and dosing intervals before T-MSCs-Exo are used clinically. Given the lack of current disease-modifying drugs, our findings highlight the role of miR-100-5p-enriched T-MSCs-Exo as a potential transformative tool in combating PD, warranting further large-scale trials and broader exploration of their impact on PD pathology.

### Supplementary Information

**Additional file 1. Fig. S1.** Typical features of T-MSCs. **Fig. S2.** T-MSCs contribute to MPP^+^-induced MN9D cell proliferation. **Fig. S3.** T-MSCs attenuate motor deficits and block α-syn aggregation in PD mice. **Fig. S4.** Identification of T-MSCs-Exo. **Fig. S5.** Enrichment and biodistribution of T-MSCs-Exo in vivo. **Fig. S6.** T-MSCs-Exo attenuate motor deficits and upregulate TH expression in PD mice. **Fig. S7.** Prediction of GO terms and statistics of GO enrichment. **Fig. S8.** miRNA-gene-GO network diagrams. **Fig. S9.** Effect of Nrf2 inhibitor ML385 treatment on the Keap1-Nrf2-SOD pathway. **Fig. S10.** Effect of inhibitor treatment on the Keap1-Nrf2-SOD pathway. **Fig. S11.** AAV-miR-100-5p attenuates motor deficits in PD mice. **Table S1.** The sequences of miR-100-5p inhibitor, inhibitor NC, miR-100-5p mimic, mimic NC. **Table S2.** The sequences of qRT-PCR primers. **Table S3. **The resource of key antibodies.

## Data Availability

The data supporting the conclusions of this article are provided in this article and the additional files. In addition, all data from this study can be obtained from the corresponding author upon reasonable request.

## Abbreviations

AAV: Adeno-associated virus
α-syn: α-Synuclein
ATCC: American Type Culture Collection
BBB: Blood–brain barrier
BCA: Bicinchoninic acid
CCK-8: Cell counting kit-8
DA: Dopaminergic
DAB: Diaminobenzidine
DCFH-DA: 2,7-Dichlorofluorescein diacetate
FCM: Flow cytometry
GSH: Glutathione
GSSG: Oxidized glutathione
hUC-MSC: Human umbilical cord MSC
IHC: Immunohistochemical staining
ISCT: International Society for Cellular Therapy
KEGG: Kyoto Encyclopedia of Genes and Genomes
MCB: Master cell bank
MSCs: Mesenchymal stem cells
MSC-Exo: MSC-derived exosomes
NC: Negative control
Nox4: NADPH oxidase 4
NTA: Nanosight tracking analysis
PD: Parkinson’s disease
PEG: Polyethylene glycol
qRT-PCR: Quantitative real-time PCR
ROS: Reactive oxyen species
SNpc: Substantia nigra pars compacta
TEM: Transmission electron microscope
TH: Tyrosine hydroxylase
T-MSCs: Trophoblast stage-derived mesenchymal stem cells
UC-MSCs: Umbilical cord mesenchymal stem cells
