# Supplementary material for: miR-100a-5p-enriched exosomes derived from mesenchymal stem cells enhance the anti-oxidant effect in a Parkinson’s disease model via regulation of Nox4/ROS/Nrf2 signaling
Source: J Transl Med. 2023 Oct 24;21:747. doi: 10.1186/s12967-023-04638-x (PMC10594913; doi:10.1186/s12967-023-04638-x)
Supplement: Supplementary file 1 — Additional file 1. Fig. S1. Typical features of T-MSCs. Fig. S2. T-MSCs contribute to MPP+-induced MN9D cell proliferation. Fig. S3. T-MSCs attenuate motor deficits and block α-syn aggregation in PD mice. Fig. S4. Identification of T-MSCs-Exo. Fig. S5. Enrichment and biodistribution of T-MSCs-Exo in vivo. Fig. S6. T-MSCs-Exo attenuate motor deficits and upregulate TH expression in PD mice. Fig. S7. Prediction of GO terms and statistics of GO enrichment. Fig. S8. miRNA-gene-GO network diagrams. Fig. S9. Effect of Nrf2 inhibitor ML385 treatment on the Keap1-Nrf2-SOD pathway. Fig. S10. Effect of inhibitor treatment on the Keap1-Nrf2-SOD pathway. Fig. S11. AAV-miR-100-5p attenuates motor deficits in PD mice. Table S1. The sequences of miR-100-5p inhibitor, inhibitor NC, miR-100-5p mimic, mimic NC. Table S2. The sequences of qRT-PCR primers. Table S3. The resource of key antibodies. [file 12967_2023_4638_MOESM1_ESM.pdf]

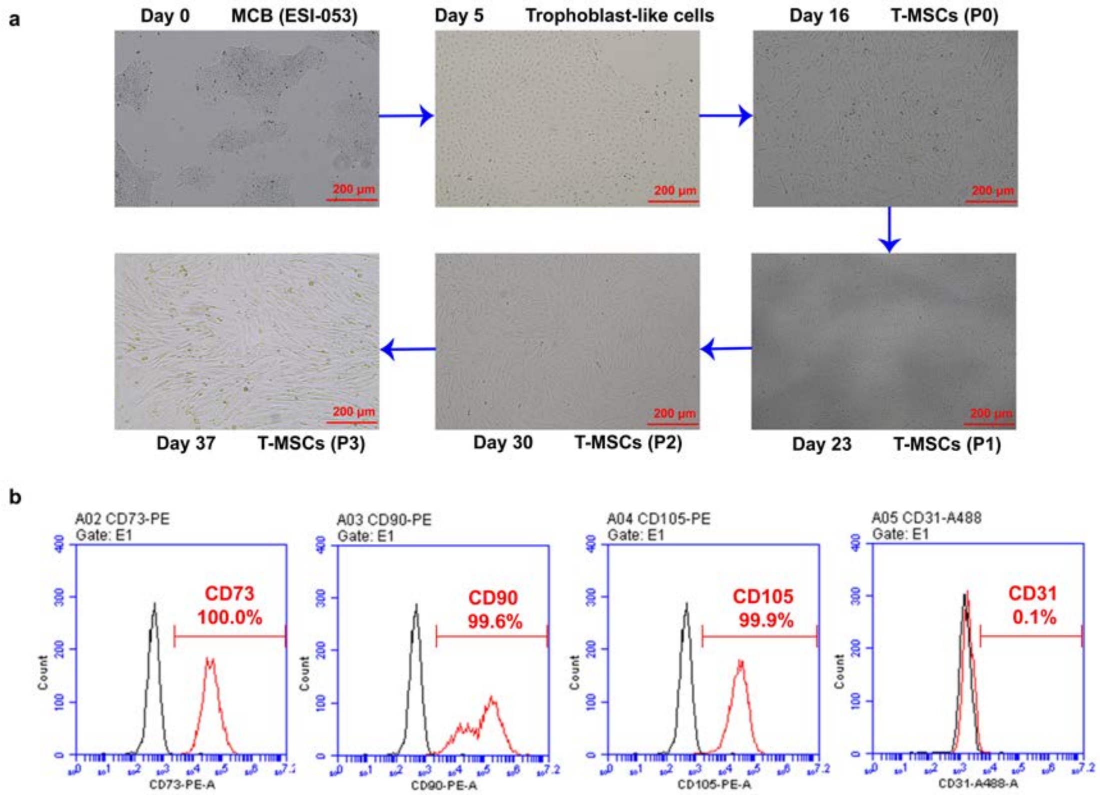

Fig. S1 Typical features of T-MSCs. a Representative micrographs showing the typical cell morphology observed during the differentiation of MCB (ESI-053) to T-MSCs. scale bar = 200 μm. **b** Phenotype of T-MSCs was analyzed by flow cytometry (black line, negative staining control; red line, specific staining for the indicated antibody). The cells were positive for CD73, CD90, and CD105 expression, and negative for CD31 expression. The percentage of cells staining positive for the indicated cell surface marker is shown on each plot.

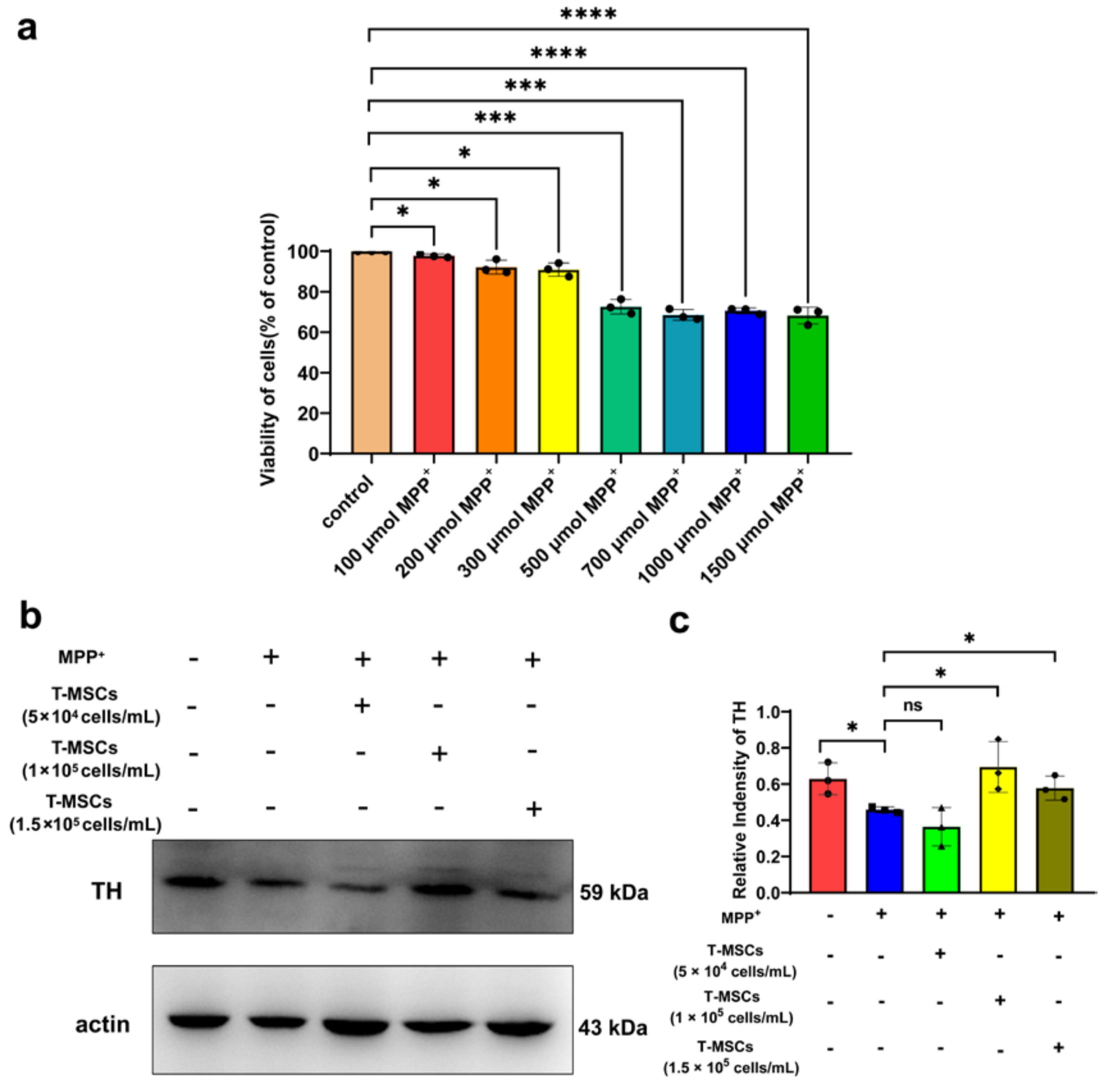

Fig. S2 T-MSCs contribute to MPP$^+$-induced MN9D cell proliferation. a CCK-8 was used to measure the MN9D cell viability after exposure to 100, 200, 300, 500, 700, 1000, and 1500 μM MPP$^+$ for 24 h. **b, c** Western blotting analysis showed the TH expression in MPP$^+$-induced MN9D cells after co-culture with different quantities of T-MSCs for 24 h. Each experiment was independently repeated three times. The results are shown as mean ± SD. One-way ANOVA was used to analyze the data. *$p < 0.05$, ***$p < 0.001$, ****$p < 0.0001$, and ns: no significant

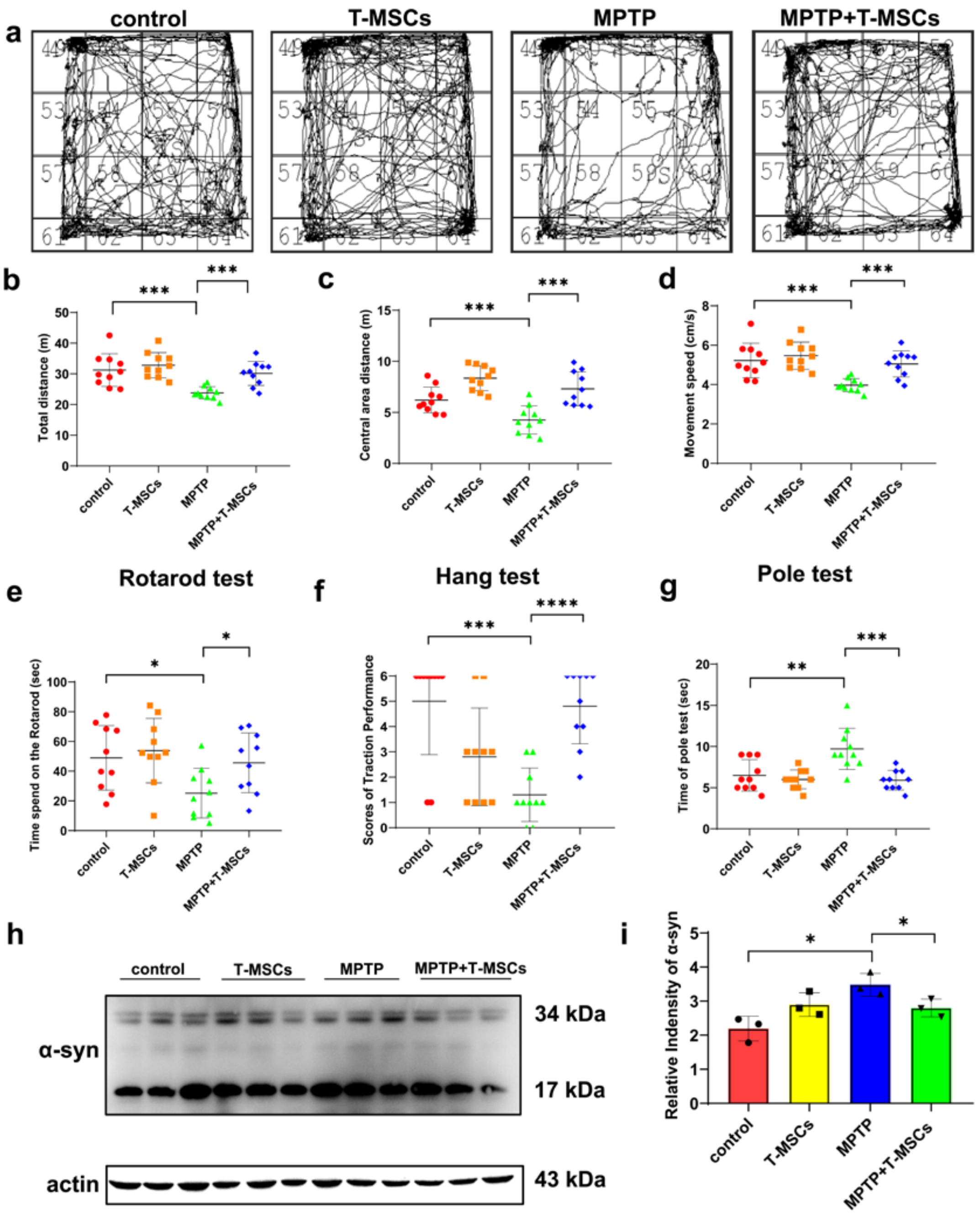

Fig. S3 T-MSCs attenuate motor deficits and block α-syn aggregation in PD mice. a-d Open field test **(a)** and the statistical plots of total distance **(b)**, central area **(c)**, and movement speed **(d)**. **e** Rotarod test. **f** Hang test. **g** Pole test. n = 10 per group. **h, i** Western blotting analysis showed the α-syn expression levels in the SN of the control, T-MSCs, MPTP, and MPTP+T-MSCs groups (n = 3 per group). The results are shown as mean ± SD. One-way ANOVA was used to analyze the data. *$p < 0.05$, **$p < 0.01$, ***$p < 0.001$, and ****$p < 0.0001$

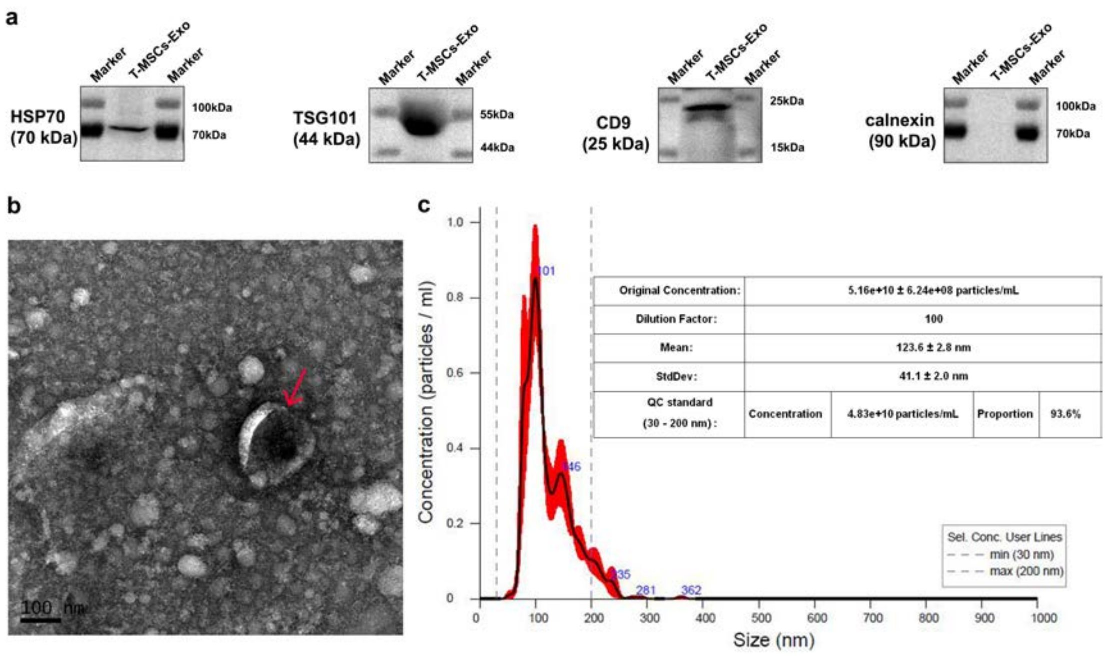

Fig. S4 Identification of T-MSCs-Exo. a Characteristics of T-MSCs-Exo surface marker proteins HSP 70, TSG101, CD9, and calnexin were analyzed by western blotting. **b** T-MSCs-Exo morphologies were observed with transmission electron microscope (TEM). Scale bars, 100 nm. **c** Size distribution and concentration of T-MSCs-Exo were measured using nanoparticle tracking analysis (NTA).

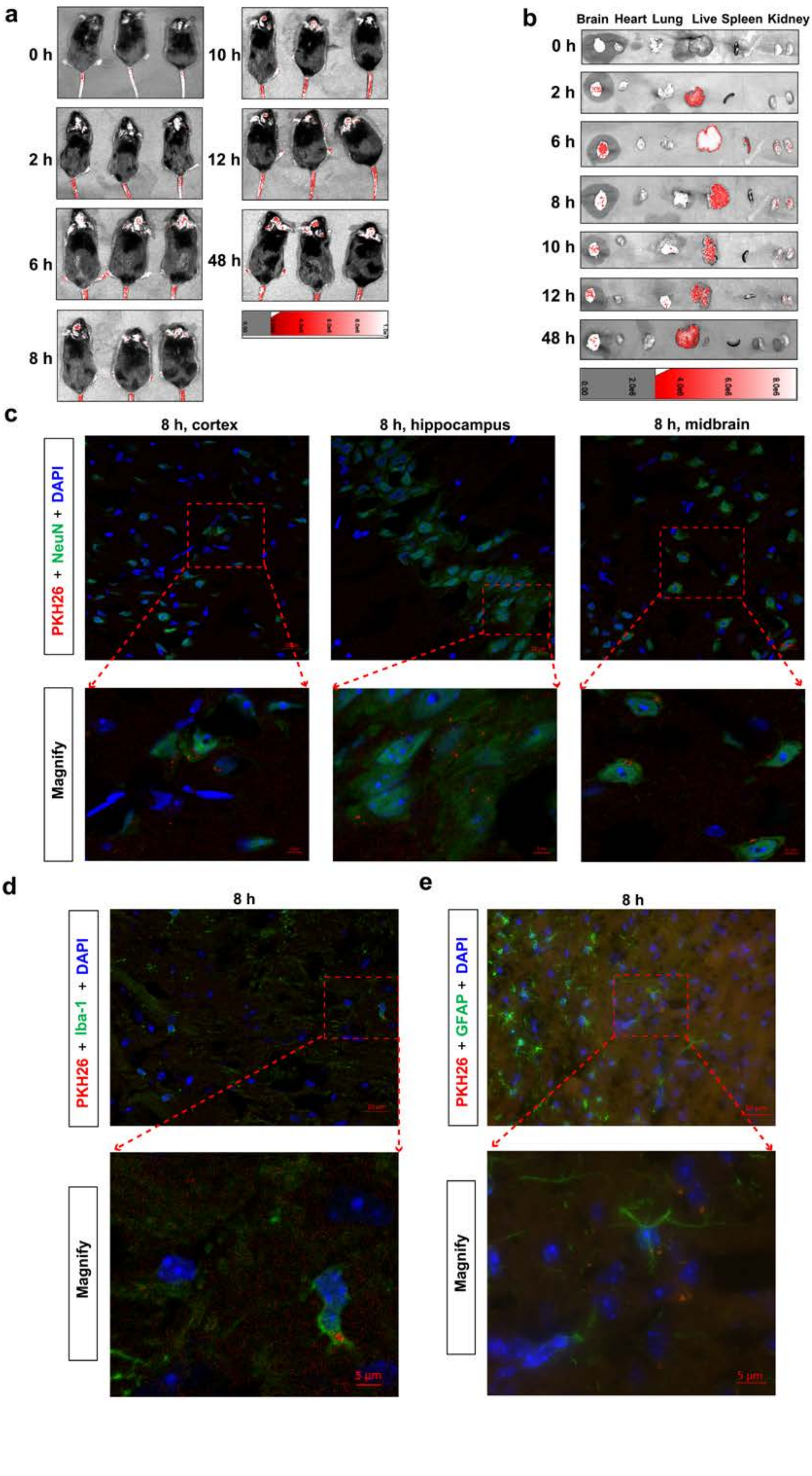

a
0 h
2 h
6 h
8 h
10 h
12 h
48 h
b
Brain Heart Lung Live Spleen Kidney
0 h
2 h
6 h
8 h
10 h
12 h
48 h
0.00
2.0e6
4.0e6
6.0e6
8.0e6
c
8 h, cortex
8 h, hippocampus
8 h, midbrain
PKH26 + NeuN + DAPI
Magnify
d
8 h
PKH26 + Iba-1 + DAPI
Magnify
5 μm
e
8 h
PKH26 + GFAP + DAPI
Magnify
5 μm

Fig. S5 Enrichment and biodistribution of T-MSCs-Exo *in vivo* . a Real time fluorescence imaging in the brain of mice detected by the Automated In-Vivo Imaging after intravenous injection of PKH26-labeled T-MSCs-Exo (n = 3 per group). **b** *Ex vivo* imaging of the sacrificed tissues after intravenous injection of PKH26-labeled T-MSCs-Exo in mice (n = 3 per group). **c** Representative immunofluorescence images of PKH26-labeled T-MSCs-Exo colocalized with neurons in three different brain regions (n = 3 per group). Scale bars, upper, 20 µm; lower, 5 µm. **d, e** Representative immunofluorescence images of PKH26-labeled T-MSCs-Exo colocalized with microglia and astrocytes in the brain regions (n = 3 per group). Scale bars, upper, 20 µm; lower, 5 µm.

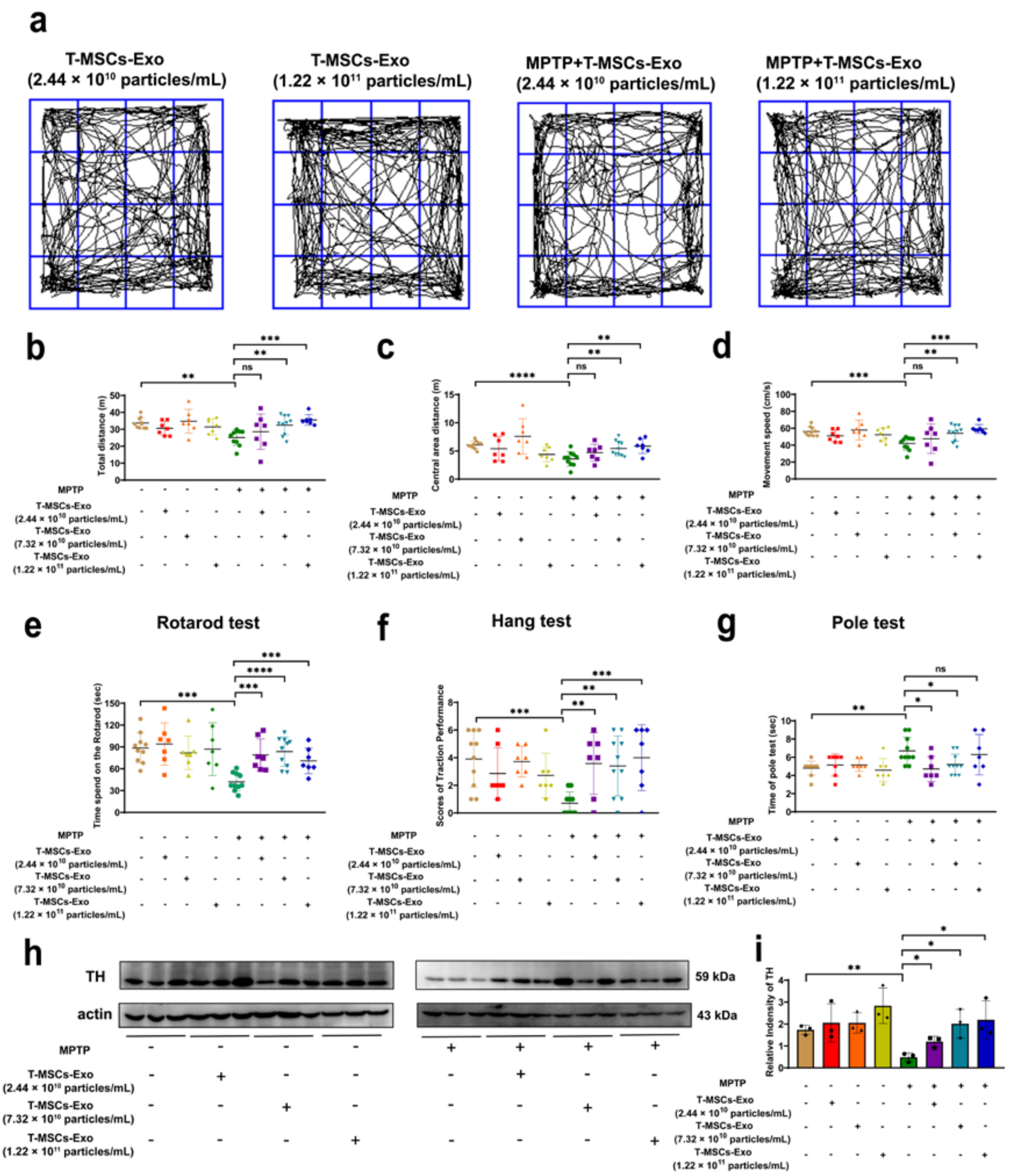

Fig. S6 T-MSCs-Exo attenuate motor deficits and upregulate TH expression in PD mice. a-d Open field test **(a)**, and its statistical plots of total distance **(b)**, central area **(c)**, and movement speed **(d)**. **e** Rotarod test. **f** Hang test. **g** Pole test. n = 10, 7, 7, 7, 10, 7,10, and 7 for the control, T-MSCs-Exo (2.44 × 10^{10} particles/mL), T-MSCs-Exo (7.32 × 10^{10} particles/mL), T-MSCs-Exo (1.22 × 10^{11} particles/mL), MPTP, MPTP+T-MSCs-Exo (2.44 × 10^{10} particles/mL), MPTP+T-MSCs-Exo (7.32 × 10^{10} particles/mL), and MPTP+T-MSCs-Exo (1.22 × 10^{11} particles/mL), respectively. **h, i** Western blotting analysis showed the TH expression levels in the SN after T-MSCs-Exo treatment of control or MPTP-induced PD mice (n = 3 per group). The

results are shown as mean ± SD. One-way ANOVA was used to analyze the data. *$p < 0.05$, **$p < 0.01$, ***$p < 0.001$, ****$p < 0.0001$, and ns: no significant

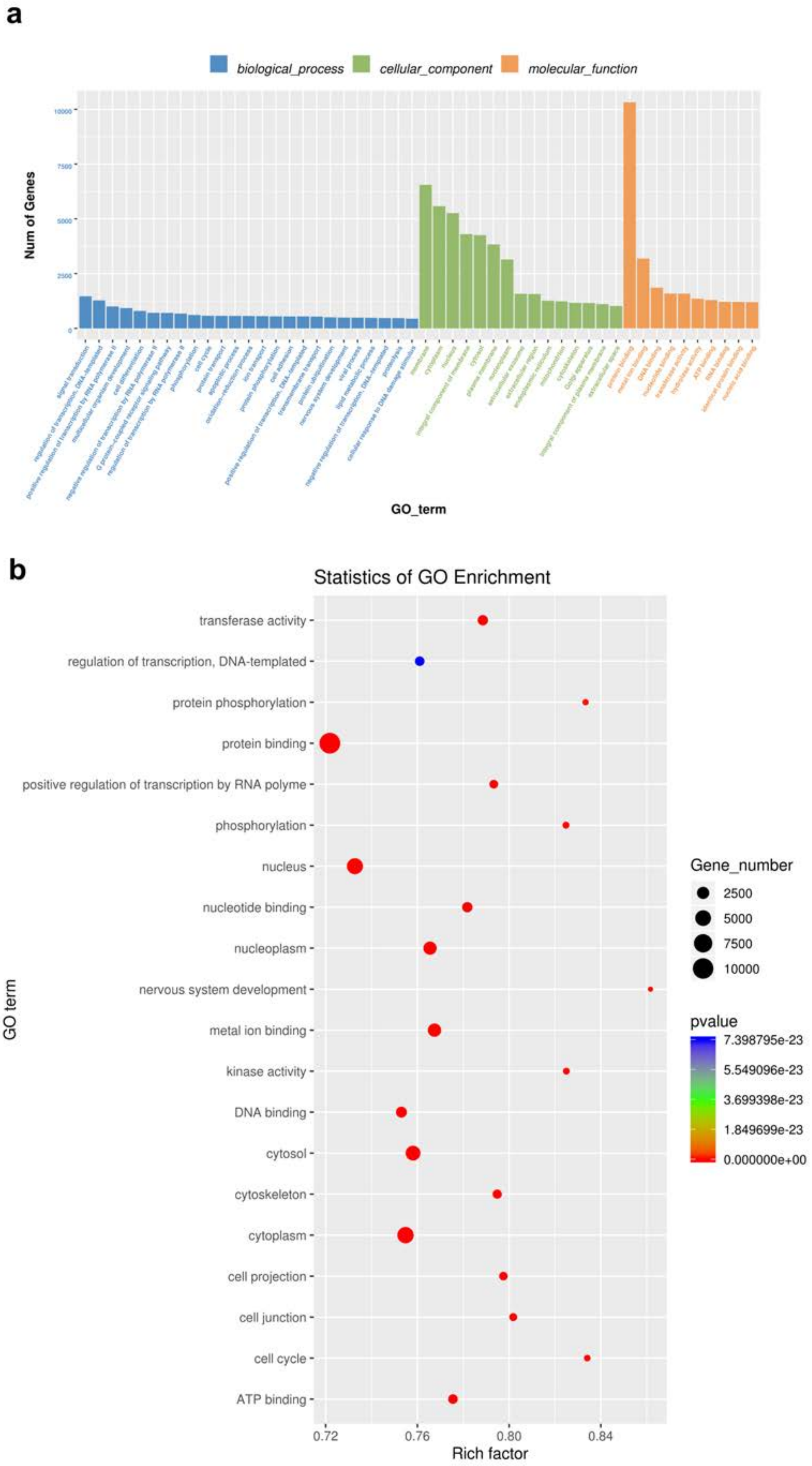

Fig. S7 Prediction of GO terms and statistics of GO enrichment.

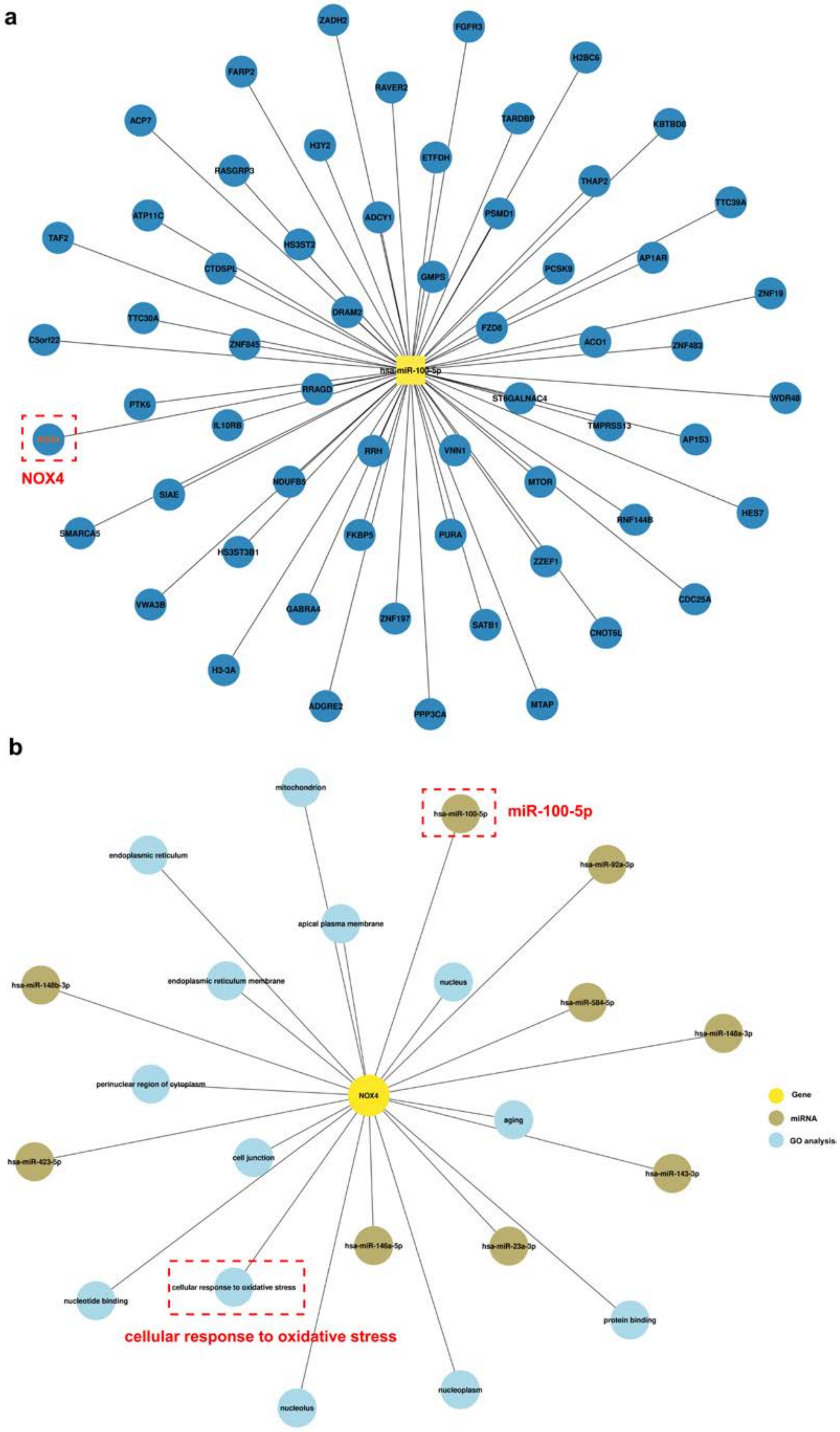

Fig. S8 miRNA-gene-GO network diagrams. **a** miR-100-5p-gene network diagram. **b** miRNAs-*NOX4*-GO network diagram.

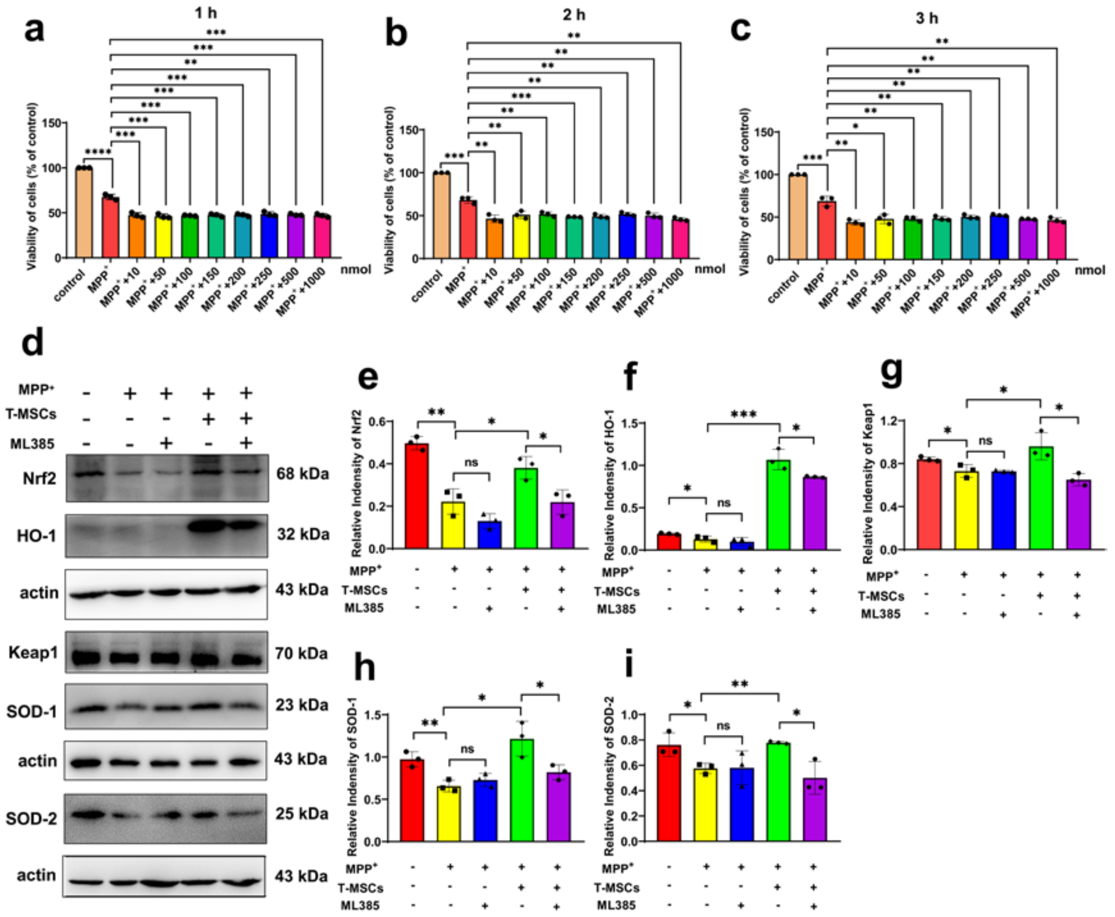

Fig. S9 Effect of Nrf2 inhibitor ML385 treatment on the Keap1-Nrf2-SOD pathway. a-c CCK-8 was used to measure the viability of MPP^+-induced MN9D cells after pretreatment with 10, 50, 100, 150, 200, 250, and 1000 nmol of Nrf2 inhibitor ML385 for 1, 2, and 3 h. **d-i** Representative blots and quantification showed the levels of Keap1, Nrf2, HO-1, SOD-1, and SOD-2 in MN9D cells, which were pre-treated with ML385 for 2 h, and co-cultured with T-MSCs for 24 h. Each experiment was independently repeated three times. The results are shown as mean ± SD. One-way ANOVA was used to analyze the data. *$p < 0.05$, **$p < 0.01$, ***$p < 0.001$, ****$p < 0.0001$, and ns: no significant

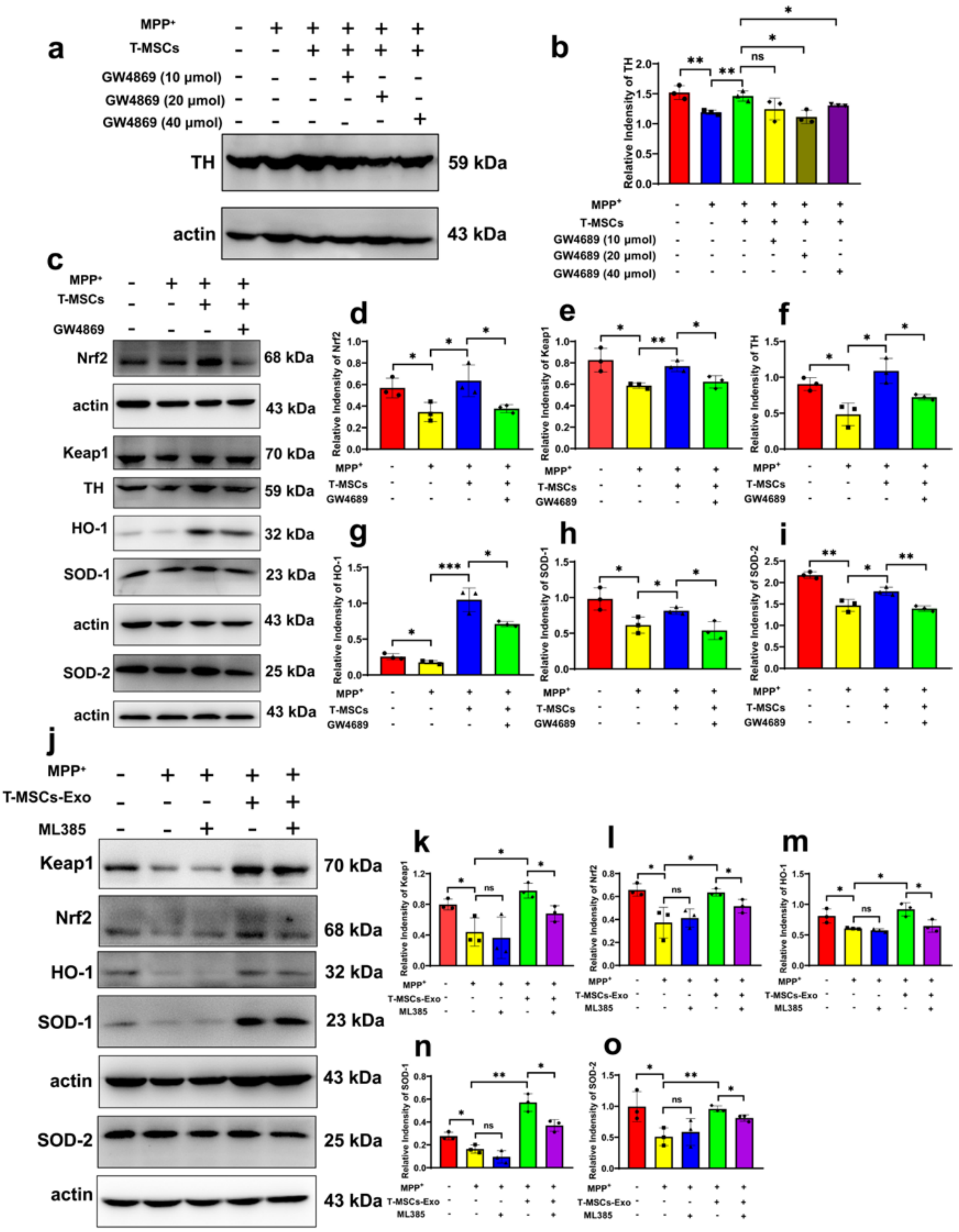

Fig. S10 Effect of inhibitor treatment on the Keap1-Nrf2-SOD pathway. a, b Western blotting analysis showed the TH expression in MN9D cells, which were pre-treated with the exosome secretion inhibitor GW4869 (10, 20, and 40 μmol) for 24 h and co-cultured with T-MSCs for 24 h. **c-i** Representative blots and quantification showed the levels of Keap1, Nrf2,TH, HO-1, SOD-1, and SOD-2 in the control, MPP^+, MPP^++T-MSCs, and MPP^++T-MSCs+GW4869 groups. **j-o** Representative blots and quantification showed the levels of Keap1, Nrf2, HO-1, SOD-1, and

SOD-2 in MN9D cells, which were pre-treated with ML385 for 2 h and treated with T-MSCs-Exo for 24 h after induction by MPP^+. Each experiment was independently repeated three times. The results are shown as mean ± SD. One-way ANOVA was used to analyze the data. *$p < 0.05$, **$p < 0.01$, ***$p < 0.001$, and ns: no significant

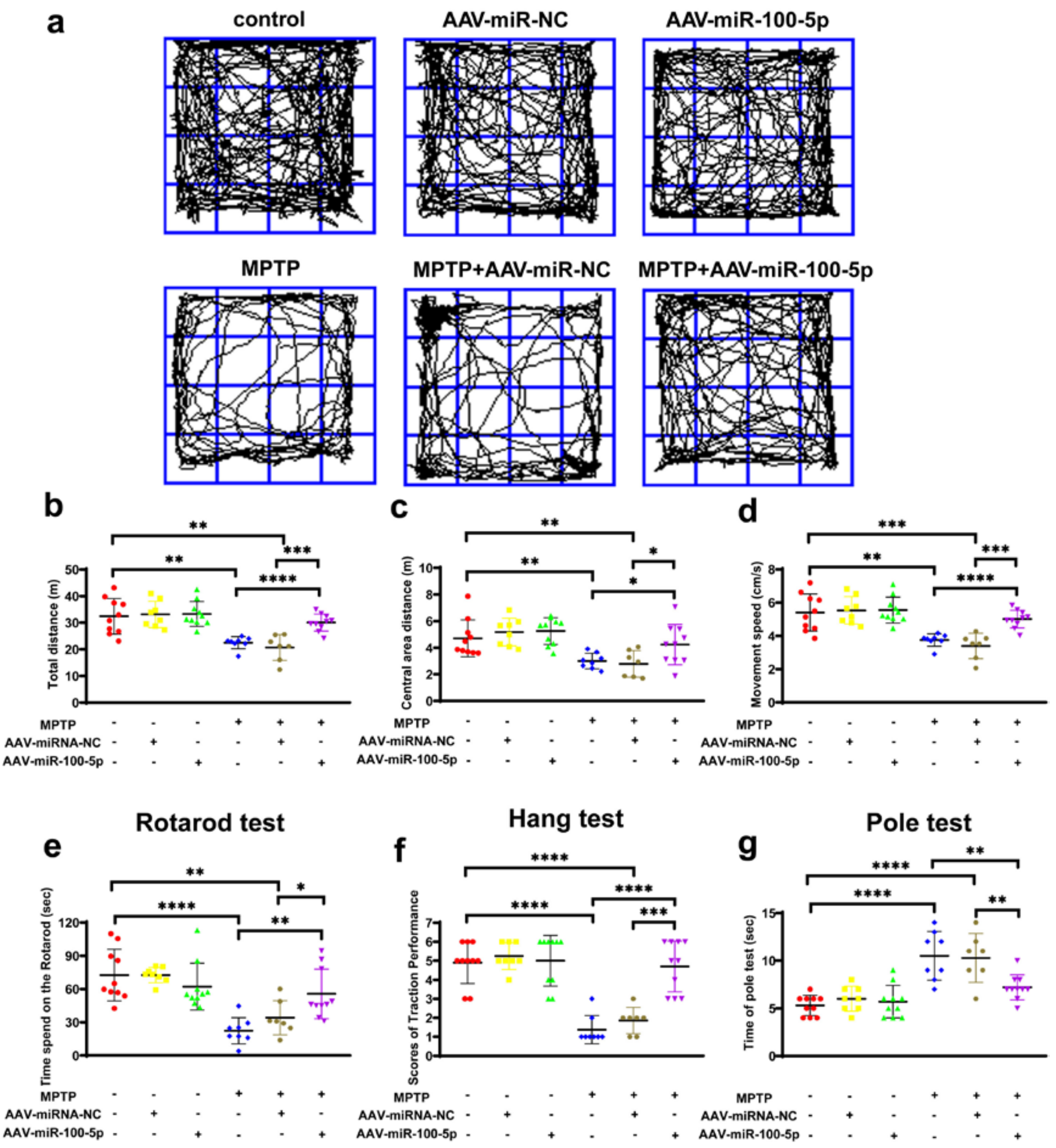

Fig. S11 AAV-miR-100-5p attenuates motor deficits in PD mice . a-d Open field test **(a)** and the associated plots of total distance **(b)**, central area **(c)**, and movement speed **(d)**. **e** Rotarod test. **f** Hang test. **g** Pole test. n = 10, 8, 10, 8, 7, and 10 for the control, AAV-miR-NC, AAV-miR-100-5p, MPTP, MPTP+AAV-miR-NC, and MPTP+AAV-miR-100-5p groups, respectively. The results are shown as mean ± SD. One-way ANOVA was used to analyze the data. **p* < 0.05, ***p* < 0.01, ****p* < 0.001, and *****p* < 0.0001

Table S1 The sequences of miR-100-5p inhibitor, inhibitor NC, miR-100-5p mimic, mimic NC

	Sense (5′-3′)	Antisense (5′-3′)
miR-100-5p inhibitor	CACAAGUUCGGAUCUACGGGUU	/
inhibitor NC	CAGUACUUUUGUGUAGUACAA	/
miR-100-5p mimic	AACCCGUAGAUCCGAACUUGUG	CAAGUUCGGAUCUACGGGUUUU
mimic NC	UUCUCCGAACGUGUCACGUTT	ACGUGACACGUUCGGAGAATT

Table S2 The sequences of qRT-PCR primers

Primer name	Sequence（5′-3′）
β-actin-F	GTGACGTTGACATCCGTAAAGA
β-actin-R	GCCGGACTCATCGTACTCC
NOX4-F	GCTACTGCCTCCATCAAGTCAAGAC
NOX4-R	CAATGCCTCCAGCCACACAGAG
miR-100-5p-F	AACAAGAACCCGTAGATCCGA
miR-100-5p-R	GTCGTATCCAGTGCAGGGT
miR-100-5p-RT	GTCGTATCCAGTGCAGGGTCCGAGGTATTCGCAC TGGATACGACCACAAGT
U6-F	CTCGCTTCGGCAGCACA
U6-R	AACGCTTCACGAATTTGCGT

Table S3 The resource of key antibodies

Antibodies	Source	Identifier
Tyrosine Hydrolase	Santa Cruz	sc-25269
α-synuclein	Santa Cruz	sc-69977
Anti-HO-1	Santa Cruz	sc-390991
Anti-SOD-1	Santa Cruz	sc-101523
Anti-SOD-2	Santa Cruz	sc-137254
Anti-HSP70	Santa Cruz	sc-24
Anti-Caspase-3	Santa Cruz	sc-7272
Anti-Keap1	Abcam	ab227828
Anti-Nrf2	Abcam	ab62352
H3	Abcam	ab1791
Anti-CD9	Abcam	ab92726
Anti-TSG101	Abcam	ab125011
Anti-Caspase-8	Abcam	ab25901
Anti- Cleaved caspase-3	Abcam	ab214430
Anti-Bax	Abcam	ab32503
Anti-Bcl-2	Abcam	ab182858
Anti-Nox4	Abcam	ab133303
Anti-Calnexin	Cell Signaling	2679
Anti-PI3K	Cell Signaling	4257T
actin	Beyotime	AA128
β-Tubulin	Beyotime	AF1216
HRP-conjugated goat anti-mouse secondary antibody	Beyotime	A0216
HRP-conjugated goat anti-rabbit secondary antibody	Beyotime	A0208
